# The meat processing exposome in Africa: integrating traditional culinary practices, environmental co-exposures, and cancer prevention strategies

**DOI:** 10.3389/fonc.2026.1749287

**Published:** 2026-04-30

**Authors:** Eugene Jamot Ndebia, Gabriel Tchuente Kamsu

**Affiliations:** School of Biological Sciences, Faculty of Medecine and Health Sciences iYunivesithi Walter Sisulu, Mthatha, South Africa

**Keywords:** African diet, cancer prevention, exposome, heterocyclicamines, one health, polycyclic aromatic hydrocarbons

## Abstract

Dietary exposure to carcinogenic compounds generated during meat processing represents a critical yet underexplored component of the African cancer exposome. Traditional high-temperature cooking methods, including smoking, grilling (braai), and singeing, are deeply embedded in African culinary culture and food security systems. However, these practices promote the formation of polycyclic aromatic hydrocarbons (PAHs), heterocyclic aromatic amines (HCAs), and N-nitroso compounds (NOCs), which undergo enzymatic bioactivation to form DNA-reactive metabolites. This chapter examines the multifaceted carcinogenic risks associated with thermally processed meats in African contexts, emphasizing the synergistic effects of dietary carcinogens and environmental co-exposures, including heavy metals and persistent organic pollutants from inappropriate fuel sources. Molecular mechanisms linking these exposures to colorectal, esophageal, hepatic, and gastric cancers are elucidated through the lens of cytochrome P450-mediated metabolism, oxidative DNA damage, and disrupted cellular signaling. Critically, this analysis demonstrates how the African exposome, characterized by the intersection of traditional food practices, environmental contamination, and socioeconomic constraints, creates unique carcinogenic exposure profiles. Evidence-based mitigation strategies are presented, including antioxidant marinades, temperature moderation, clean fuel adoption, and improved smoking technologies. These interventions must be implemented within a One Health framework that integrates food safety policy, community engagement, and environmental health protection. By reconciling traditional culinary heritage with contemporary cancer prevention science, this chapter charts a pathway toward culturally respectful yet health-protective dietary practices across African communities.

## 1 Introduction: situating meat processing within the African cancer exposome

### 1.1 The exposome concept: from genome to environment

The exposome paradigm, introduced by Wild (1) and refined over two decades, encompasses the totality of environmental exposures from conception onward, including dietary, chemical, biological, physical, and psychosocial factors that interact dynamically with genetic architecture to influence disease risk (2, 3). This conceptual framework emerged from recognition that while genomics explains a substantial portion of disease heritability, environmental determinants account for the majority of chronic disease burden, particularly cancers. Unlike the genome, which remains relatively static, the exposome is highly dynamic and potentially modifiable, offering actionable targets for primary prevention.

Within the exposome framework, diet occupies a unique position: it simultaneously serves as a source of essential nutrients, bioactive compounds with chemopreventive potential, and chemical contaminants with carcinogenic properties (4, 5). In African populations, where infectious diseases historically dominated the epidemiological landscape, the cancer burden has escalated dramatically over recent decades, a transition partly attributable to changing dietary patterns, urbanization, and persistent environmental exposures (6).

Understanding cancer risk in Africa, therefore, requires examining how traditional food practices intersect with broader environmental, occupational, and behavioral exposure dimensions. Among dietary exposures, the thermal processing of animal-source foods through smoking, grilling, singeing, and curing represents a particularly critical yet underexplored component of the African cancer exposome, generating multiple carcinogenic compound classes simultaneously within culturally embedded practices that affect millions of consumers daily.

### 1.2 Traditional food processing as a convergence point of multiple exposures

Traditional meat and fish processing methods in Africa, particularly smoking, open-flame grilling, and singeing, represent a convergence point where dietary, environmental, and occupational exposures intersect. These techniques, developed over centuries to extend food preservation in resource-limited settings without refrigeration, are deeply woven into the socioeconomic and cultural fabric of African communities (7). Smoking and grilling enhance palatability, develop characteristic flavors, reduce moisture content to inhibit microbial growth, and create economically accessible protein sources for both urban and rural populations.

However, these same processes generate a complex mixture of thermogenic carcinogens. When organic matter, whether animal fats, proteins, or fuel sources, undergoes incomplete combustion at temperatures exceeding 200 °C, polycyclic aromatic hydrocarbons (PAHs) form through pyrolytic reactions (8). Simultaneously, the Maillard reaction between amino acids, creatine, and reducing sugars produces heterocyclic aromatic amines (HCAs) (9). Additional risks arise from endogenous formation of N-nitroso compounds (NOCs), catalyzed by heme iron in red meat reacting with nitrites or nitrates (10). The formation of N-nitroso compounds during meat processing has received renewed regulatory attention following the European Food Safety Authority’s comprehensive risk assessment, which concluded that exposure to N-nitrosamines from dietary sources raises a health concern for all age groups across the European population (11). Concurrently, the risk-benefit paradigm for thermally processed red meat has gained prominence, recognizing that heating processes simultaneously generate toxic compounds and modify nutritional parameters in ways requiring integrated evaluation (12).

Critically, the African food processing exposome extends beyond the food matrix itself. Many traditional practices employ fuel sources that introduce exogenous contaminants: used motor oil, discarded tires, plastic waste, and low-quality charcoal contribute heavy metals (lead, cadmium, mercury, arsenic), dioxins, furans, and persistent organic pollutants (13, 14). These co-exposures create synergistic genotoxic effects that amplify cancer risk beyond what would be predicted from dietary carcinogens alone (15). Workers in informal meat-processing sectors face additional occupational exposures through chronic smoke inhalation and dermal contact with contaminated surfaces.

### 1.3 Operationalizing the meat processing exposome

**Figure 1:**
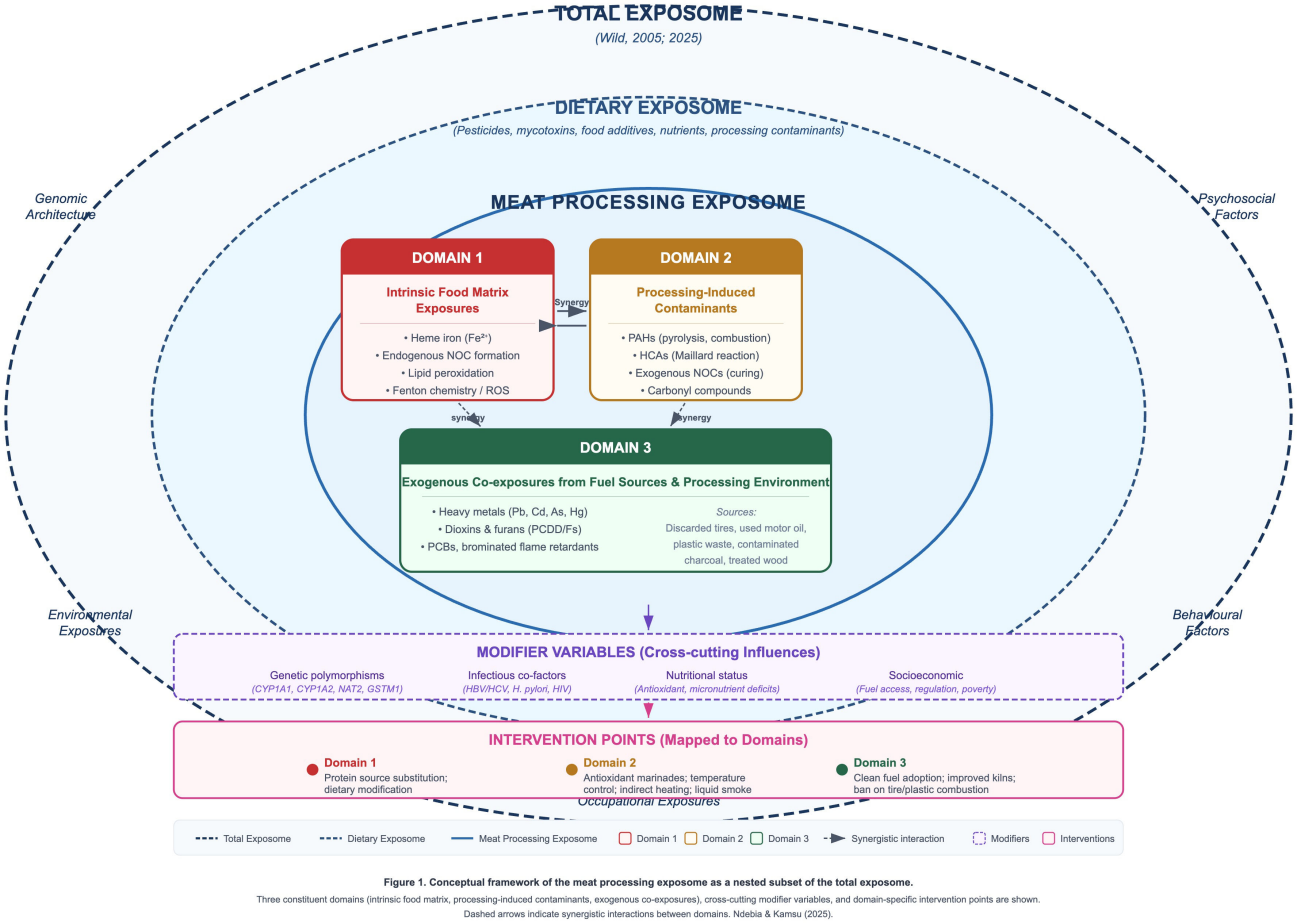
Conceptual framework of the meat processing exposome as a nested subset of the total exposome. Three constituent domains (intrinsic food matrix, processing-induced contaminants, exogenous co-exposures), cross-cutting modifier variables, and domain-specific intervention points are shown. Dashed arrows indicate synergistic interactions between domains.

For the purposes of this chapter, we define the meat processing exposome as the totality of chemical exposures arising directly from or mediated by the thermal processing of animal-source foods, encompassing three hierarchically organized domains (**Figure 1**).

Domain 1 — Intrinsic food matrix exposures: Endogenous compounds inherent to the raw food substrate that become toxicologically activated during digestion or metabolism, principally heme iron and its catalytic role in endogenous N-nitroso compound formation and lipid peroxidation (16).

Domain 2 — Processing-induced contaminants: Chemical species generated *de novo* during thermal treatment through pyrolysis, incomplete combustion, and Maillard chemistry, including polycyclic aromatic hydrocarbons (PAHs), heterocyclic aromatic amines (HCAs), and exogenous N-nitroso compounds (NOCs) from curing processes (8, 9).

Domain 3 — Exogenous co-exposures from fuel sources and environment: Contaminants introduced not by the food itself but by the processing environment, particularly heavy metals (lead, cadmium, arsenic, mercury), persistent organic pollutants (dioxins, furans, PCBs, brominated flame retardants), and additional PAH congeners derived from inappropriate fuel sources such as discarded tires, used motor oil, and plastic waste (13, 14).

This tripartite framework distinguishes the meat processing exposome from broader constructs in two critical respects. First, unlike the total dietary exposome, which encompasses all food-borne chemical exposures including pesticide residues, mycotoxins, food additives, and naturally occurring toxicants, the meat processing exposome is restricted to exposures causally linked to thermal processing of animal tissues. Second, unlike the general environmental exposome, which includes ambient air pollution, water contaminants, and occupational hazards across all settings, our framework captures environmental contaminants only insofar as they enter the human body through the food processing pathway — whether by deposition on food surfaces, absorption into the food matrix, or occupational inhalation during processing activities.

The operational value of this framework lies in its identification of modifiable intervention points. Each domain corresponds to distinct prevention strategies: Domain 1 exposures are mitigated through protein source substitution or dietary modification; Domain 2 through cooking method optimization (temperature control, marination, indirect heating); and Domain 3 through fuel substitution and improved processing technology. By disaggregating the meat processing exposome into these domains, researchers and policymakers can prioritize interventions based on relative contribution to total carcinogenic burden and feasibility of implementation in specific African contexts.

### 1.4 Chapter scope and objectives

This chapter examines carcinogenic implications of meat processing practices within African food systems through an exposome lens. We synthesize current knowledge on comparative intrinsic and processing-related cancer risks across animal-source foods, chemical formation pathways for dietary carcinogens, regional cooking traditions and their exposure profiles, environmental co-exposures amplifying toxicity, and molecular mechanisms linking thermogenic compounds to carcinogenesis. The analysis prioritizes African contexts, highlighting how socioeconomic constraints, fuel availability (defined here as the type, quality, and affordability of combustion materials accessible to food processors, ranging from clean liquefied petroleum gas (LPG) in urban commercial settings to discarded tires and plastic waste in informal slaughter facilities), regulatory gaps, and cultural practices shape exposure patterns. We evaluate scientifically validated mitigation strategies adaptable to traditional practices and propose policy frameworks integrating cancer prevention into food safety and environmental health systems. Ultimately, we demonstrate that reducing cancer risk requires not abandoning traditional practices, but evolving them through scientific knowledge, appropriate technology, and community-engaged implementation consistent with One Health principles.

## 2 Comparative carcinogenic potential of animal-source foods

### 2.1 IARC classification framework: red meat, processed meat, and fish

The International Agency for Research on Cancer (IARC) classification system provides the authoritative framework for evaluating carcinogenic hazards. Based on comprehensive reviews of epidemiological, experimental, and mechanistic evidence, IARC classifies red meat (mammalian muscle tissue including beef, pork, lamb, veal, mutton, and goat) as probably carcinogenic to humans (Group 2A) (17, 18). This classification reflects robust evidence of increased colorectal cancer risk with consumption, along with suggestive associations with pancreatic and prostate cancers.

Processed meat, defined as meat preserved through salting, curing, fermentation, smoking, or other processes to enhance flavor or improve preservation, is classified as carcinogenic to humans (Group 1), the same category as tobacco smoke and asbestos (19). This classification applies to products including bacon, ham, hot dogs, sausages, corned beef, and smoked or cured meats. The distinction between red meat and processed meat is critical: while unprocessed red meat presents carcinogenic risks primarily through heme iron content and formation of compounds during high-temperature cooking, processed meats carry additional risks from curing agents (nitrites/nitrates) and smoking-derived contaminants.

For fish and poultry, no intrinsic carcinogenic classification exists. The low heme iron content in white meat (poultry) and the favorable omega-3 fatty acid profile in many fish species generally confer a lower baseline risk compared to red meat (20). However, carcinogenic hazards emerge primarily through processing methods: smoking, grilling, and frying introduce PAHs and HCAs regardless of the protein source (21). Thus, a grilled chicken breast or smoked fish may present comparable or even greater carcinogenic exposure than a gently cooked beef stew, highlighting the critical importance of cooking method in determining final risk.

### 2.2 Intrinsic risk factors: heme iron, lipid composition, and protein structure

#### 2.2.1 Heme iron and endogenous nitrozation

Heme iron (Fe²^+^), abundant in red meat myoglobin, functions as a key endogenous risk factor through multiple pathways.

First, heme catalyzes formation of N-nitroso compounds in the gastrointestinal lumen through nitrozation of dietary amines and amides (22, 23). This process is particularly pronounced in the colon, where heme reaches high concentrations and interacts with luminal contents. Nitrozylated heme iron, formed when nitrites (whether from processed meat additives or endogenous nitric oxide) bind to heme demonstrates five to six times greater genotoxic potency than non-nitrozylated heme (24). Recent advances in analytical chemistry now enable precise quantification of nitrosyl-heme pigments in cured meat products using high-performance liquid chromatography with diode-array and fluorescence detection (HPLC-DAD-FLD), facilitating exposure assessment for this particularly potent genotoxic species (25).

Second, heme iron generates reactive oxygen species (ROS) through Fenton chemistry, promoting lipid peroxidation of colonocyte membranes. The resulting aldehydic products including malondialdehyde and 4-hydroxynonenal form DNA adducts and stimulate compensatory colonocyte hyperproliferation, creating a cellular environment conducive to neoplastic transformation (16).

Third, heme disrupts the colonic epithelial barrier, increasing permeability to luminal carcinogens and promoting chronic low-grade inflammation.

Poultry and fish contain substantially lower heme iron concentrations (approximately 10-20% of levels in beef), partially explaining their lower intrinsic carcinogenic risk (26). However, this relative advantage is diminished when these proteins undergo high-temperature processing that generates exogenous carcinogens.

#### 2.2.2 Lipid composition and thermal degradation

Lipid content and fatty acid composition profoundly influence carcinogen formation during cooking. Red meat typically contains 15-30% fat by weight with high proportions of saturated fatty acids, which undergo extensive pyrolysis when exposed to temperatures exceeding 200 °C (27). Fat dripping onto heat sources generates dense smoke laden with PAHs; these volatile compounds then deposit onto food surfaces. High-fat cuts consequently accumulate significantly greater PAH burdens than lean cuts prepared identically.

The positive correlation between lipid content and PAH formation has been consistently demonstrated across multiple food matrices: studies in pork products show that samples with >20% fat content generate 2- to 4-fold higher B[a]P concentrations than lean cuts (<10% fat) prepared identically (12, 27). Similar findings have been reported for grilled poultry, where skin-on preparations (containing 12–15% fat) produce significantly higher PAH4 levels than skinless preparations (28). Fish species with elevated lipid content, such as mackerel and sardine (8–15% fat), accumulate more PAHs during smoking than lean species like tilapia (2–4% fat), even under identical processing conditions (29).

The scenario differs for fish rich in polyunsaturated fatty acids (PUFAs), particularly omega-3 fatty acids (eicosapentaenoic acid, EPA; docosahexaenoic acid, DHA). While PUFAs confer cardiovascular benefits and potential anti-inflammatory effects, they are more susceptible to thermal oxidation than saturated fats, potentially generating different toxic lipid peroxidation products during high-temperature cooking (30). Paradoxically, the health benefits of omega-3 fatty acids may be partially offset when oily fish undergo prolonged smoking or high-temperature grilling.

Poultry occupies an intermediate position: skinless white meat contains relatively little fat (2-5%), minimizing pyrolysis-derived PAHs, but skin and dark meat harbor higher fat concentrations (10-15%) that approach red meat levels when cooked with skin intact (31).

#### 2.2.3 Protein structure and HCA formation

All muscle tissues contain the biochemical precursors necessary for HCA formation: free amino acids (particularly phenylalanine, glutamic acid, and lysine), creatine/creatinine (abundant in mammalian muscle), and reducing sugars. However, creatine concentrations vary substantially across species: red meat contains approximately 4–5 g/kg, poultry 3–4 g/kg, and fish generally less than 2 g/kg (9). This gradient partially explains why beef and pork consistently generate higher HCA levels than fish when cooked under identical conditions.

The most thoroughly studied HCA, 2-amino-1-methyl-6-phenylimidazo[4,5-b]pyridine (PhIP), demonstrates clear mutagenic properties in bacterial assays and mammary carcinogenicity in rodent models. However, the relevance of these animal findings to human cancer risk remains under investigation, with epidemiological evidence suggestive but not definitive for breast and colorectal cancers (24).

### 2.3 Nutritional value versus carcinogenic risk: a public health paradox

The carcinogenic potential of red meat, processed meat, and thermally treated animal proteins exists in tension with their nutritional contributions. Red meat provides highly bioavailable heme iron (absorption rate 15-35% compared to 2-20% for non-heme iron from plant sources), essential for preventing iron-deficiency anemia, a persistent public health problem across Africa, particularly affecting women and children (26). Red meat also supplies complete protein with optimal amino acid profiles, vitamin B_12_ (absent from plant sources), zinc, selenium, and other micronutrients critical for growth, immune function, and neurological development.

For many African communities, particularly in resource-limited settings, meat and fish represent irreplaceable sources of high-quality protein and micronutrients. Smoked fish, for instance, provides more than 60% of dietary protein in some West and Central African regions, serving as a nutritional cornerstone (32, 33). Similarly, traditional meat products like *suya*, *kilichi*, and *chichinga* provide economically accessible protein that would otherwise be unattainable for low-income urban populations.

This creates a genuine public health dilemma: recommendations to reduce consumption of processed and red meat, while epidemiologically sound for cancer prevention, risk exacerbating malnutrition, anemia, and protein deficiency in populations where these foods fill critical nutritional gaps. The solution lies not in eliminating these foods, but in reducing carcinogenic exposures through improved processing methods while maintaining their nutritional accessibility a theme developed throughout subsequent sections.

The tension between nutritional benefits and carcinogenic risks of thermally processed meats has catalyzed the development of formal risk-benefit assessment (RBA) frameworks in food safety science. The European Food Safety Authority and other regulatory bodies increasingly advocate integrated evaluations that simultaneously quantify health benefits (nutrient supply, bioavailability) and health risks (carcinogen exposure, chronic disease endpoints) within a common metric, typically disability-adjusted life years (DALYs) (11, 12). In African contexts, such frameworks would need to account for the disproportionate nutritional dependence on animal-source foods in populations with limited dietary alternatives, the higher baseline burden of protein-energy malnutrition and micronutrient deficiencies, and the substantially elevated contaminant levels arising from traditional processing methods relative to industrial production. No formal risk-benefit assessment integrating these African-specific parameters has yet been published, representing a significant methodological gap. Such analyses would provide evidence-based foundations for nuanced dietary guidelines that optimize the nutritional contributions of processed meats while minimizing carcinogenic exposure, thereby replacing blanket prohibitions with targeted improvements in processing (34).

## 3 Chemistry and formation pathways of dietary carcinogens

### 3.1 Polycyclic aromatic hydrocarbons: combustion products and lipid pyrolysis

#### 3.1.1 Chemical structure and formation mechanisms

Polycyclic aromatic hydrocarbons comprise a family of organic compounds containing two or more fused aromatic rings. Their formation during food processing occurs through two principal mechanisms: incomplete combustion of organic materials (fuel sources) and pyrolysis of food components, particularly lipids and proteins (35). Both processes require temperatures typically exceeding 400 °C and occur most extensively under oxygen-limited conditions that promote formation of carbon-centered free radicals (34, 35), which subsequently condense into aromatic ring structures.”

More than 100 distinct PAH compounds have been identified in food, but risk assessment typically focuses on a subset with established carcinogenic properties. Benzo[a]pyrene (B[a]P) serves as the reference compound due to extensive toxicological characterization and classification as a Group 1 human carcinogen by IARC. The European Union regulates PAHs in food through Commission Regulation (EU) 2023/915, which repealed and replaced Regulation (EC) No 1881/2006. Both individual benzo[a]pyrene (B[a]P) and PAH4 — the sum of benzo[a]pyrene, benzo[a]anthracene, benzo[b]fluoranthene, and chrysene — are subject to maximum limits. For smoked meat and fish products, the maximum permitted levels are 2 μg/kg for B[a]P and 12 μg/kg for PAH4 (36). Additional focus extends to PAH8 and PAH16 profiles for comprehensive exposure assessment (29, 34, 35).

To contextualize these regulatory thresholds against African realities: B[a]P concentrations in traditionally smoked catfish from Nigeria range from 122–288 μg/kg (30, 37), exceeding the EU maximum limit of 12 μg/kg for smoked products (EU Regulation 2023/915) by factors of 10–24. In contrast, fish processed using improved Chorkor kilns in Ghana typically yield B[a]P levels of 8–15 μg/kg (38), approaching but occasionally still exceeding regulatory thresholds, underscoring both the severity of the problem and the potential for technological mitigation.

#### 3.1.2 Deposition pathways in traditional cooking

During smoking and grilling, PAH contamination occurs through multiple simultaneous routes. Smoke generated from incomplete fuel combustion carries vaporized PAHs that condense on food surfaces as temperature gradients develop. The extent of deposition depends on smoke density, contact duration, surface moisture (which influences compound solubility), and food lipid content (PAHs preferentially partition into fatty matrices due to their lipophilic nature). Smoked products, therefore accumulate PAHs predominantly in outer layers and fatty portions.

A second critical pathway involves lipid pyrolysis: when meat fat drips onto hot coals or heating elements, it ignites or smolders, generating dense smoke particularly enriched in high-molecular-weight PAHs. This smoke rises and deposits on the underside of food positioned above the heat source, a phenomenon especially pronounced in traditional open-flame grilling, where meat is positioned directly above coals. Studies demonstrate that this dripping mechanism contributes more to total PAH burden than direct smoke deposition in many grilling scenarios (28, 31).

#### 3.1.3 Fuel source influence on PAH profiles

The type of fuel dramatically shapes both total PAH burden and specific congener profiles. Hardwood combustion (common in traditional smoking) generates moderate PAH levels with profiles dominated by pyrogenic PAHs formed during combustion. Charcoal, particularly low-quality charcoal with residual volatile matter, produces substantially higher PAH concentrations, reaching levels that can exceed regulatory limits even with brief cooking times (39).

African studies reveal particularly concerning exposures when non-food materials serve as fuel sources. In West African contexts where meat singeing employs used automobile tires, PAH concentrations in treated meat exceed European limits by factors of 10-50 (13). Tire combustion releases not only classical PAHs but also rubber-derived additives and aromatic compounds not typically encountered in food contexts, complicating risk assessment. Similarly, burning plastic waste introduces chlorinated and brominated PAH derivatives with potentially enhanced toxicity.

### 3.2 Heterocyclic aromatic amines: the Maillard reaction at high temperatures

#### 3.2.1 Formation chemistry

Heterocyclic aromatic amines arise through a distinct mechanism from PAHs, forming via Maillard chemistry, the reaction cascade between amino acids and reducing sugars that also generates desirable color and flavor compounds in cooked foods. However, when the Maillard reaction proceeds at temperatures exceeding 150 °C, particularly in the presence of creatine or creatinine (abundant in muscle tissue), it can divert toward HCA formation (8).

The two principal HCA classes differ in formation temperature and pathways. Aminoimidazoazarenes (including PhIP and MeIQx) form at moderate temperatures (150-250 °C) through reactions between creatine, amino acids, and hexoses. Aminocarbolines (including AαC and MeAαC) require higher temperatures (>300 °C) and arise from pyrolysis of amino acids and proteins. Consequently, grilling and pan-frying generate primarily aminoimidazoazarenes, while smoking at very high temperatures can produce both classes.

Quantitative data on HCA concentrations in African food products remain limited compared to PAH literature. Available studies report PhIP concentrations in Nigerian suya (grilled beef) ranging from 12–28 ng/g (37), comparable to levels documented in well-done grilled beef in European and North American studies (10–30 ng/g) (9). MeIQx concentrations in grilled meats from Egyptian markets reach 8–15 ng/g (40). These values should be interpreted against the absence of internationally harmonized regulatory limits for HCAs in food, a regulatory gap reflecting ongoing scientific debate about dose-response relationships at typical dietary exposure levels.

#### 3.2.2 Factors governing HCA formation

Multiple variables influence HCA formation kinetics. Temperature shows a strong positive relationship: concentrations increase exponentially above 175 °C, explaining why well-done meat contains dramatically more HCAs than rare or medium preparations. Cooking time also matters, though the relationship is complex, initial formation accelerates rapidly, but prolonged cooking may partially degrade some HCAs through further thermal reactions.

Moisture content exerts protective effects: wet cooking methods (boiling, stewing, steaming) maintain food temperatures below 100 °C and produce negligible HCA levels. Even in dry cooking, maintaining surface moisture through marinades or basting reduces HCA formation. This occurs because water acts as a heat sink, limiting surface temperature, and because hydration affects Maillard reaction kinetics.

Food matrix composition determines HCA precursor availability. Red meat, with its high creatine content, generates more HCAs per unit surface area than fish cooked identically. However, the absolute amounts depend on cooking method: thinly sliced fish grilled at very high temperatures may accumulate more total HCAs than thick beef steaks cooked gently due to greater surface area exposure relative to mass.

### 3.3 N-nitroso compounds: endogenous and exogenous formation routes

N-nitroso compounds (NOCs) represent a broad and chemically diverse class united by the N–N=O functional group, encompassing N-nitrosamines, N-nitrosamides, and other subclasses. It is important to note that the well-established carcinogenic potency of NOCs is primarily attributable to volatile N-nitrosamines, particularly N-nitrosodimethylamine (NDMA) and N-nitrosodiethylamine (NDEA) rather than to the entire NOC class uniformly (11). Throughout this chapter, we use ‘NOCs’ when referring to the broad compound class formed during endogenous nitrozation and ‘N-nitrosamines’ when discussing specific carcinogenic species and their risk assessment.

#### 3.3.1 Exogenous NOCs in processed meat

N-nitroso compounds represent a diverse chemical class united by the N-N=O functional group. In processed meats, NOCs form when nitrite or nitrate curing salts (added for color stabilization, flavor development, and antimicrobial protection) react with secondary amines naturally present in muscle tissue. This reaction occurs spontaneously under acidic conditions typical of cured meat products, accelerated by heat during cooking.

The most toxicologically significant NOCs are N-nitrosamines, several of which demonstrate potent carcinogenic properties in animal models at concentrations as low as parts per billion. N-nitrosodimethylamine (NDMA) and N-nitrosodiethylamine (NDEA) have received particular scrutiny due to their presence in various processed meats and demonstrated mutagenic activity (41).

Modern meat processing employs various strategies to minimize NOC formation while maintaining curing benefits: reducing nitrite concentrations to minimum effective levels (often 50–150 ppm), adding antioxidants (ascorbic acid, erythorbic acid, α-tocopherol) that compete with nitrosation reactions, and optimizing pH and temperature profiles during processing. However, these controls are rarely implemented in informal meat processing sectors common across Africa, where traditional empirical methods dominate.

Quantitative NOC data from African processed meat products are extremely scarce. Nabizadeh et al. (41) reported NDMA concentrations of 1.2–8.7 μg/kg in processed meat products from Middle Eastern markets, which share some processing similarities with North African practices. South African commercially processed meats (polony, Vienna sausages) have not been systematically assessed for NOC content in published literature, representing a critical data gap given the high consumption of these products in lower-income urban communities (24).

#### 3.3.2 Endogenous NOC formation in the gastrointestinal tract

The endogenous nitrosation pathway represents an equally or more important contributor to total NOC exposure, particularly for unprocessed red meat. In this scenario, dietary heme iron catalyzes nitrosation reactions in the acidic stomach environment and the large intestine. The reaction substrates include dietary amines from various protein sources, endogenous amines produced by gut microbiota, and nitrogen oxides derived from swallowed saliva (which contains nitrate reduced to nitrite by oral bacteria) or endogenous nitric oxide production.

This endogenous formation pathway explains why even consumption of fresh, unprocessed red meat increases fecal NOC levels in controlled feeding studies, an effect not observed with poultry or fish consumption (10). The magnitude of endogenous NOC formation correlates positively with heme iron intake, providing mechanistic support for the epidemiological association between red meat consumption and colorectal cancer.

Nitrosylated heme, a distinct species formed when nitric oxide binds to heme iron, deserves particular mention. This compound demonstrates remarkably potent genotoxic effects in colonic epithelial cells, operating through mechanisms distinct from classic NOCs: it generates reactive oxygen species, lipid peroxidation products, and DNA-reactive aldehydes at concentrations five- to sixfold lower than required for equitoxic effects from non-nitrosylated heme (16). Nitrosylated heme forms both during industrial processing of cured meats and endogenously when heme iron encounters nitric oxide in the gut.

### 3.4 Expected contaminant levels attributable to cooking methods: a summary

To provide a consolidated reference, **Table 1** summarizes expected mean concentration ranges for the three principal carcinogen classes generated during thermal processing, stratified by cooking method. These ranges derive from controlled experimental studies and market surveys predominantly from European, Asian, and African settings. These values demonstrate that cooking method exerts a far greater influence on contaminant burden than protein source, reinforcing the chapter’s central argument that modifying processing practices rather than eliminating traditional foods represents the most effective and culturally appropriate prevention strategy.

**Table 1:** Expected concentration ranges of major carcinogens by cooking method.

Compound class	Boiling / stewing	Pan-frying (moderate)	Grilling (charcoal, direct flame)	Traditional smoking (6–24 h)	Tire/plastic singeing
B[a]P (μg/kg)	<0.1 (negligible)	0.1–2.0	1.0–67	8–288	387–892
PAH4 (μg/kg)	<0.5	0.5–8.0	4–156	42–450	>1,000
PhIP (ng/g)	<0.1	1–15	5–28	<2 (low temp)	Not characterised
MeIQx (ng/g)	<0.1	0.5–8	2–15	<1	Not characterised
NDMA (μg/kg)	<0.5	<1.0	1–5 (if cured meat)	1–8 (if nitrite used)	Not characterised
Total volatile N-nitrosamines (μg/kg)	<1.0	1–5	2–12	3–15	Not characterised

Compiled from Pinto da Costa et al. (9), EFSA CONTAM Panel (11), Iammarino et al. (12), Sampaio et al. (35), Adeyeye & Ashaolu (37), Darwish et al. (40), and African contamination surveys reviewed in Section 4.4.

## 4 Traditional African meat processing: cultural practices and exposure profiles

### 4.1 Socioeconomic significance of smoking, grilling, and singeing

Traditional meat and fish processing methods in Africa serve functions extending far beyond simple food preservation. These practices constitute fundamental economic activities providing livelihoods for millions, particularly women in rural communities who process and market smoked fish (42). The techniques preserve protein-rich foods in tropical climates lacking reliable refrigeration infrastructure, extending shelf life from days to weeks or months and enabling distribution from production areas to distant markets.

Culturally, these foods carry profound significance. Grilled meat preparations, *suya* in Nigeria and Ghana, *nyama choma* in East Africa, *braai* in Southern Africa, *kilichi* in Sahelian regions transcend mere sustenance to function as social glue at celebrations, religious festivals, and community gatherings (37). The distinctive flavors, aromas, and textures developed through smoking and grilling are integral to food identity and culinary heritage. Proposals to abandon these practices would encounter not merely practical resistance but cultural opposition rooted in identity and tradition.

Economically, processed meat products provide accessible protein for urban populations. Smoked fish retails at prices 20-40% below fresh fish, while street-vended grilled meat offers affordable portions unattainable with formal market pricing (24). For low-income households, these products represent realistic access to animal-source nutrition essential for child growth and maternal health.

### 4.2 Regional cooking methods: fuel types, temperatures, and contamination patterns

Traditional meat and fish processing methods vary substantially across African sub-regions, shaped by ecological conditions, protein sources, cultural heritage, and economic infrastructure. The evidence base, however, is unevenly distributed: West Africa (particularly Nigeria and Ghana) and Southern Africa (South Africa) account for the majority of published contamination studies, while Central Africa, East Africa, and North Africa remain substantially under-represented in the peer-reviewed literature (**Table 2**). This asymmetry reflects both research infrastructure disparities and funding priorities rather than absence of risk. Where regional data are sparse, we extrapolate cautiously from comparable practices documented elsewhere, noting explicitly when doing so.

**Table 2:** Regional summary of traditional meat processing practices, fuel sources, and contamination data across african sub-regions.

Sub-region	Dominant practices	Primary fuel sources	Key contaminants documented	Evidence base
West Africa (Nigeria, Ghana, Benin, Côte d'Ivoire, Cameroon)	Fish smoking (Chorkor/traditional kilns); meat grilling (*suya*, *kilishi*, *chichinga*); carcass singeing	Hardwood, charcoal, discarded tires, used motor oil, plastic waste	PAHs (B[a]P 18–892 μg/kg); heavy metals (Pb, Cd, As); dioxins	Extensive: Multiple contamination surveys, risk assessments, intervention studies
East Africa (Kenya, Ethiopia, Tanzania, Uganda)	Grilling (*nyama choma*); smoking (Nile perch, tilapia); roasting	Hardwood, charcoal, agricultural residues	PAHs (B[a]P 14–98 μg/kg); limited heavy metal data	Moderate: Selected studies on smoked fish and grilled meats; limited biomonitoring
Southern Africa (South Africa, Mozambique, Zimbabwe)	Open-flame grilling (*braai*); processed meat consumption (boerewors, polony)	Charcoal, hardwood, gas (urban)	PAHs (B[a]P 0.2–41 μg/kg); NOCs from processed meats; HCAs	Moderate to extensive: Well-characterized braai exposures; limited data from rural areas
Central Africa (DRC, Congo, Gabon, Equatorial Guinea)	Fish smoking; bushmeat smoking; singeing	Hardwood, mangrove wood, waste fuels in urban areas	Limited quantitative data; expected high PAH contamination based on methods	Sparse: Very few peer-reviewed contamination studies; critical research gap
North Africa (Egypt, Morocco, Tunisia, Algeria)	Grilling (*kofta*, *kebab*, *mechoui*); oven roasting	Charcoal, wood, gas	PAHs (B[a]P in grilled meat); limited smoking-related data	Limited: Egyptian studies on grilled meat; minimal data from Maghreb countries

#### 4.2.1 West and Central Africa: smoking dominance

Smoking predominates as the primary fish preservation method across coastal and riverine West and Central Africa, with estimates suggesting 60-70% of caught fish undergoes smoking before marketing (33). Traditional smoking kilns vary regionally but share common features: an enclosed or semi-enclosed structure where fish hangs above a smoldering fire for 6–24 hours. Fuel sources include various hardwoods, particularly abundant species like *Acacia auriculiformis*, cocoa wood, and oil palm residues.

These extended smoking durations and enclosed geometries maximize smoke contact, generating substantial PAH deposition. Studies across Ghana, Nigeria, Benin, and Côte d’Ivoire consistently document B[a]P concentrations in smoked catfish (*Clarias gariepinus*), tilapia (*Oreochromis niloticus*), and sardinella (*Sardinella maderensis*) ranging from 50 to 288 μg/kg, levels 4 to 24 times above EU regulatory thresholds (30, 38). Hot smoking, which combines smoke exposure with heat sufficient to cook tissue (temperatures 70-90 °C), produces particularly high contamination relative to cold smoking techniques used in some European contexts.

Recent interventions introduced improved kiln designs, particularly the Chorkor oven (Ghana) and FTT-Thiaroye processing unit (Senegal), which separate combustion and smoking chambers, control airflow to improve combustion efficiency, and maintain lower processing temperatures (43). Adoption studies demonstrate 40-60% reduction in PAH contamination compared to traditional kilns, though uptake remains limited by higher capital costs and technical training requirements.

#### 4.2.2 Southern and East Africa: grilling traditions

Open-flame grilling, *braai* in Afrikaans, represents the dominant traditional cooking method across Southern Africa, with comparable practices (*nyama choma*) throughout East Africa. Unlike smoking, which emphasizes preservation, grilling focuses on immediate consumption, generating distinctive charred surfaces and smoky flavors highly valued in regional cuisines.

Traditional grilling employs direct heat, positioning meat 5–15 cm above wood or charcoal fires reaching 400-900 °C (31, 39). Fat dripping onto hot coals ignites or smolders, creating dense smoke that deposits PAHs on the meat underside. This cooking geometry and the high surface temperatures promote both PAH deposition and HCA formation through Maillard chemistry (31).

Contamination levels vary substantially with grilling parameters. Studies of South African braai practices document B[a]P ranging from 0.2 μg/kg in gas-grilled chicken to 40 μg/kg in charcoal-grilled pork sausages (boerewors) (24). Prolonged grilling to achieve well-done preparations particularly amplifies risk: PhIP concentrations in well-done beef (internal temperature >77 °C, heavily browned crust) can exceed 20 ng/g compared to <1 ng/g in lightly cooked (rare; internal temperature ~52–55 °C, minimal surface browning) preparations (8).

The apartheid legacy and socioeconomic disparities in Southern Africa create distinct exposure patterns. Lower-income urban communities show greater reliance on inexpensive processed meats (polony, Vienna sausages) with residual nitrite concentrations typically ranging from 30–150 mg/kg (as sodium nitrite), approaching or reaching the maximum permitted levels in South African regulations (200 mg/kg NaNO_2_ as ingoing amount) (11, 12). These products accumulate risks from both N-nitrosamine formation during thermal preparation and PAH exposure during grilling or frying.

### 4.3 Meat singeing with non-food fuels: a critical exposure pathway

#### 4.3.1 The practice and its cultural context

Meat singeing (*flambage* in francophone contexts) constitutes a distinct processing step employed primarily for beef, goat, and pork carcasses across West Africa and parts of East Africa (44). The practice involves direct flame application to carcass skin to burn off hair and surface debris, simultaneously creating a roasted surface layer. For pork specifically, singeing softens and partially crisps skin, creating texture desirable in regional cuisines.

This technique emerged from practical necessity: it represents the most time-efficient method for processing large carcasses in slaughter facilities lacking hot water systems or mechanical dehairers. In Nigeria, Ghana, Cameroon, and other West African countries, singeing is nearly universal at slaughterhouses and wet markets processing ruminants and pigs (13).

#### 4.3.2 The critical problem: non-food fuel sources

The toxicological crisis arises from fuel choices. Proper singeing employs clean propane burners or torches fueled by liquefied petroleum gas (LPG). However, acquisition costs for these devices and ongoing LPG expenses prove prohibitive for many small-scale butchers. Consequently, readily available waste materials serve as alternatives: discarded automobile tires, used motor oil, plastic waste, and petroleum-soaked rags predominate in informal slaughter contexts (14).

Tire combustion generates extraordinarily complex chemical mixtures. Vulcanized rubber contains not only hydrocarbon polymers but also heavy metals (zinc, lead, cadmium from vulcanization catalysts), aromatic process oils, plasticizers, and flame retardants. Incomplete combustion releases these materials as particulates and volatiles that condense on meat surfaces. Studies of tire-singed meat document PAH concentrations 10 to 50 times higher than EU limits, with particularly elevated levels of high-molecular-weight PAHs (benzo[g,h,i]perylene, indeno[1,2,3-c,d]pyrene) that exhibit greater carcinogenic potency than B[a]P (13).

Plastic combustion introduces additional hazards: dioxins, furans, polychlorinated biphenyls (PCBs), and brominated flame retardants, all persistent organic pollutants with endocrine-disrupting and carcinogenic properties. Used motor oil adds petroleum-derived PAHs, heavy metals (lead from older formulations), and partially oxidized hydrocarbons.

#### 4.3.3 The public health urgency of this practice cannot be overstated

Tire and plastic combustion for meat singeing constitutes an illegal practice under food safety legislation in virtually all African countries where it occurs, yet enforcement remains negligible due to inadequate inspection capacity, corruption, and the economic marginality of informal slaughter operations. The resulting contamination levels B[a]P concentrations exceeding EU limits by 32- to 74-fold, heavy metals surpassing Codex thresholds by factors of 80–400 represent among the most extreme food safety violations documented globally in any food category.

Unlike other carcinogenic exposures discussed in this chapter that require nuanced risk-benefit evaluation, tire and plastic singeing offers no legitimate trade-off: cleaner alternatives (LPG torches, clean wood fires) achieve identical processing outcomes at modest additional cost. The continued practice reflects a failure of governance, not an absence of solutions. Every carcass singed with tire combustion exposes not only consumers to extreme carcinogenic contamination but also processing workers to chronic inhalation of one of the most toxic smoke mixtures documented in occupational health literature a mixture classified by its individual components as containing multiple IARC Group 1 carcinogens.

We therefore recommend that the elimination of tire and plastic combustion in meat singeing be designated as the highest priority food safety intervention in the African meat processing sector, pursued through coordinated regulatory enforcement, subsidized access to LPG equipment, and public awareness campaigns targeting both producers and consumers. This single intervention would achieve greater reduction in population carcinogenic exposure than any other measure discussed in this chapter.

#### 4.3.4 Heavy metal co-contamination

Beyond PAHs, heavy metals represent a critical co-exposure pathway in singed and smoked products. Studies across Ghana and Nigeria document lead concentrations in singed beef reaching 2.7–43.7 mg/kg, cadmium 0.4–19.5 mg/kg, and arsenic 0.2–12.3 mg/kg. These values vastly exceed both Codex Alimentarius limits (Pb: 0.10 mg/kg; Cd: 0.050 mg/kg in meat) and European Union maximum levels established under Commission Regulation (EU) 2023/915 (Pb: 0.10 mg/kg in meat of bovine animals, sheep, pig, and poultry; Cd: 0.050 mg/kg in meat excluding offal) by factors of 27–437 for lead and 8–390 for cadmium (36). These metals originate from fuel contaminants and deposit on meat surfaces through smoke particulates.

Heavy metal exposure from contaminated foods operates through distinct toxicological mechanisms from PAHs: lead disrupts heme synthesis and neurodevelopment, cadmium accumulates in kidney and bone causing renal dysfunction and osteoporosis, arsenic induces oxidative DNA damage and epigenetic alterations, and mercury affects neurological development particularly in fetuses (45). When combined with PAH and HCA exposures, these metals create multiplicative carcinogenic risk through synergistic oxidative stress mechanisms discussed in Section 5.

### 4.4 Quantifying contamination: PAH and heavy metal levels in African food systems

#### 4.4.1 Regional contamination surveys

Systematic contamination surveys across African markets reveal widespread exceedances of regulatory thresholds. A comparative reference table that juxtaposes African contamination levels against EU, Codex, and US EPA benchmarks is summarized in **Table 3**. A comprehensive review of studies from 2000–2024 spanning 15 African countries documents:

**Table 3:** Comparison of contaminant concentrations in african processed meats against international regulatory benchmarks.

Contaminant	African product (range)	EU maximum limit	Codex alimentarius limit	US EPA reference	Exceedance factor
B[a]P	Smoked fish, Nigeria (122–288 μg/kg)	12 μg/kg (smoked fish)	12 μg/kg	—	10–24×
B[a]P	Tire-singed beef, Ghana (387–892 μg/kg)	12 μg/kg	12 μg/kg	—	32–74×
B[a]P	Braai meats, South Africa (0.2–41 μg/kg)	12 μg/kg	12 μg/kg	—	0.02–3.4×
PAH4	Smoked tilapia, Ghana (85–156 μg/kg)	12 μg/kg (smoked fish)	—	—	7–13×
Lead (Pb)	Singed meat, Ghana/Nigeria (2.7–43.7 mg/kg)	0.10 mg/kg (meat)	0.10 mg/kg	—	27–437×
Cadmium (Cd)	Singed meat, Cameroon (4–19 mg/kg)	0.050 mg/kg (meat)	0.05 mg/kg	—	80–380×
Arsenic (As)	Smoked/singed products (0.2–12.3 mg/kg)	—	—	0.003 mg/kg/day (oral RfD)	Context-dependent
PhIP	Suya, Nigeria (12–28 ng/g)	No established limit	No established limit	No established limit	No benchmark

Smoked Fish:

- Nigerian smoked catfish (2017–2020): B[a]P 122–288 μg/kg (30, 37)
- Ghanaian smoked tilapia (2018–2021): PAH4 85–156 μg/kg (38)
- Benin smoked sardinella (2017–2019): B[a]P 68–142 μg/kg (42)
- Kenya smoked Nile perch (2017): PAH4 42–98 μg/kg (30)

Grilled Meat:

- Nigerian suya, beef (2020): B[a]P 18–67 μg/kg, PhIP 12–28 ng/g (37)
- South African braai, mixed meats (2016): B[a]P 4–41 μg/kg (24)
- Ethiopian grilled lamb (2022): B[a]P 14–36 μg/kg (46)

Singed Meat:

- Ghana tire-singed beef (2023–2025): B[a]P 387–892 μg/kg (13)
- Nigeria tire-singed goat (2024): PAH16 total 1,240–2,856 μg/kg (14)

These data demonstrate that traditional processing in current practice generates carcinogen exposures substantially exceeding levels considered acceptable in international food safety frameworks (13, 30, 37, 38). However, exposure assessment requires contextualizing these concentrations within actual consumption patterns.

#### 4.4.2 Exposure and risk assessment

Cancer risk assessment employs the Incremental Lifetime Cancer Risk (ILCR) framework, which integrates contaminant concentrations, consumption rates, body weight, exposure duration, and compound-specific cancer potency factors. ILCR values above 10^−4^ (one additional cancer case per 10,000 exposed individuals over a lifetime) are generally considered unacceptable from public health perspectives (35, 47).

Studies applying this framework to African contexts reveal concerning findings:

- Calabar, Nigeria (smoked fish consumption): ILCR = 2.99 × 10^−6^ (acceptable but elevated) (48)
- Egypt (grilled meat): ILCR = 1.86 × 10^−5^ (18× benchmark level) (40)
- Urban Ghana (mixed diet including smoked fish and grilled meat): ILCR = 4.2 × 10^−5^ (42× benchmark) (38)

Interpreting these ILCR estimates requires careful contextualization to avoid overstating risk or stigmatizing cultural practices. Several considerations are essential:

First, consumption frequency and portion size profoundly influence actual exposure. An individual consuming smoked fish once weekly faces substantially lower cumulative PAH exposure than a daily consumer, potentially by an order of magnitude. Population consumption data from West Africa indicate that while smoked fish contributes significantly to overall protein intake, daily consumption of heavily contaminated products is limited to specific occupational groups and high-frequency consumers rather than representing population-wide behavior (32).

Second, comparative risk framing is essential for proportionate public health messaging. The ILCR estimates documented above (10^−6^ to 10^−4^ range) should be interpreted alongside other modifiable cancer risk factors prevalent in African populations: tobacco smoking (relative risk 15–30 for lung cancer), heavy alcohol consumption (RR 2–5 for upper aerodigestive cancers), hepatitis B/C infection without treatment (RR 20–100 for hepatocellular carcinoma), and physical inactivity (RR 1.2–1.5 for colorectal cancer). While dietary carcinogen exposure from processed meats represents a genuine and modifiable risk, it is typically of smaller magnitude than these major established risk factors for the same cancers in most consumption scenarios (6).

Third, the risk is in the method, not the food. A central message of this chapter is that the carcinogenic hazard derives primarily from specific processing conditions — extreme temperatures, inappropriate fuels, prolonged smoke contact — rather than from the cultural practice of smoking or grilling per se. The same foods prepared with modified techniques (lower temperatures, clean fuels, antioxidant marinades) yield contaminant levels within or near acceptable ranges, as demonstrated by improved kiln studies (38). This distinction is fundamental to culturally respectful public health communication.

These estimates likely underrepresent true risk for several reasons: they typically assess single compound or compound class exposures without accounting for synergistic effects between PAHs, HCAs, NOCs, and heavy metals; they assume average consumption patterns whereas high consumers face disproportionate risk; and they do not incorporate other exposome dimensions (air pollution, occupational exposures, infectious co-factors) that modify cancer susceptibility.

## 5 Environmental co-exposures and synergistic carcinogenic effects

### 5.1 Heavy metals in smoked and grilled foods: sources and health implications

#### 5.1.1 Contamination pathways

Heavy metal contamination of processed animal-source foods in African contexts arises through three distinguishable but frequently co-occurring pathways, each requiring distinct intervention strategies:

Pathway A — Fuel-source contamination (primary contributor for singed products): The combustion of discarded tires, used motor oil, plastic waste, and chemically treated wood introduces metals directly into smoke that deposits on food surfaces. Tire vulcanization employs zinc oxide, lead-based stabilizers, and cadmium-containing pigments; their combustion releases these metals as particulates and volatile species. This pathway accounts for the most extreme contamination levels documented in the literature: lead concentrations of 2.7–43.7 mg/kg and cadmium of 0.4–19.5 mg/kg in tire-singed beef (13, 49). Critically, this pathway is entirely eliminable through fuel substitution.

Pathway B — Environmental pre-contamination of raw animal tissue: Animals bioaccumulate metals from contaminated environments before slaughter or harvest. Fish from industrially polluted waterways accumulate mercury (through methylmercury biomagnification), cadmium, and lead in muscle and organ tissues (15). Livestock grazing near mining operations, industrial facilities, or waste dumps similarly accumulate metals. These pre-existing burdens concentrate during drying and smoking as moisture loss reduces tissue mass without eliminating metals. This pathway is mitigated through environmental regulation and food safety monitoring of raw materials.

Pathway C — Food contact materials and processing infrastructure: Metal contamination may also arise from contact with galvanized wire grills (zinc), painted or corroded metal drying racks (lead from older paints, iron oxides), and recycled metal containers used during processing. While less extensively documented than Pathways A and B, studies from Nigerian fish markets report elevated nickel concentrations (7.7–149 mg/kg) potentially attributable to nickel-plated drying racks (15). This pathway is addressed through infrastructure improvement and use of food-grade materials.

The relative contribution of each pathway varies by product type and regional context. For tire-singed meat, Pathway A dominates overwhelmingly. For conventionally smoked fish, Pathways B and C typically contribute more substantially, though Pathway A may contribute when low-quality charcoal containing waste materials is used. Disaggregating these pathways is essential for intervention design: eliminating tire combustion (Pathway A) would dramatically reduce contamination of singed products but would not address pre-existing environmental contamination in fish (Pathway B) or infrastructure-related exposure (Pathway C).

#### 5.1.2 Molecular mechanisms of metal toxicity

Specific heavy metals classified as known human carcinogens (IARC Group 1: arsenic and inorganic arsenic compounds, cadmium and cadmium compounds, chromium(VI) compounds, nickel compounds) or probable/possible human carcinogens (Group 2A: lead and inorganic lead compounds; Group 2B: methylmercury) operate through mechanisms complementary to but distinct from organic carcinogens like PAHs. Cadmium, arsenic, chromium(VI), and nickel induce carcinogenesis through:

1. Oxidative DNA Damage: Metals generate reactive oxygen species through Fenton-type reactions, directly damaging DNA bases and creating strand breaks
2. Enzyme Inhibition: Metals bind to sulfhydryl groups in phase II detoxification enzymes (glutathione S-transferases, superoxide dismutase), compromising cellular defense mechanisms
3. Epigenetic Alterations: Arsenic and cadmium modify DNA methylation patterns and histone acetylation, dysregulating tumor suppressor gene expression
4. DNA Repair Disruption: Metals interfere with nucleotide excision repair and base excision repair pathways, allowing mutations to persist
5. Immune Suppression: Chronic metal exposure impairs immune surveillance of pre-neoplastic cells

These mechanisms operate synergistically with PAH-induced DNA adducts: when repair capacity is compromised by metals, PAH-DNA lesions persist longer, increasing mutation probability. Population-based studies demonstrate that combined high-level exposures to both PAHs and heavy metals produce cancer risks exceeding the sum of individual risks, true synergism rather than simple additivity (6, 14, 15).

### 5.2 Persistent organic pollutants from inappropriate fuel sources

#### 5.2.1 Chemical classes and formation

The combustion of plastics, treated wood, painted materials, and petroleum products generates persistent organic pollutants (POPs) that accumulate in processed foods and human tissues. Key compound classes include:

Polychlorinated Dibenzo-p-dioxins (PCDDs) and Dibenzofurans (PCDFs): Formed when chlorine-containing materials (PVC plastic, chlorinated pesticide residues, bleached paper) combust in the presence of organic matter and catalytic metals. The most toxic congener, 2,3,7,8-tetrachlorodibenzo-p-dioxin (TCDD), is a Group 1 human carcinogen with potency exceeding that of most PAHs by several orders of magnitude.

Polychlorinated Biphenyls (PCBs): Though banned internationally, PCBs persist in electrical equipment, old building materials, and contaminated soils. Incomplete combustion of contaminated materials releases PCBs into smoke that deposits on food.

Brominated Flame Retardants (BFRs): Polybrominated diphenyl ethers (PBDEs) and related compounds from burning electronic waste, upholstery foam, and plastic casings produce brominated analogues of dioxins with similar toxicological properties.

These compounds share characteristic features: high lipophilicity (preferential accumulation in fatty tissues), resistance to metabolic degradation (resulting in bioaccumulation and biomagnification), and environmental persistence (half-lives measured in years to decades) (13, 14, 50).

#### 5.2.2 Toxicological consequences

POPs exert carcinogenic effects primarily through aryl hydrocarbon receptor (AhR) activation. Upon binding dioxins or dioxin-like PCBs, AhR translocates to the nucleus and induces expression of cytochrome P450 enzymes (particularly CYP1A1, CYP1A2, CYP1B1) (51). While this response initially represents an adaptive detoxification attempt, chronic activation creates multiple carcinogenic liabilities:

1. Sustained CYP1A1 overexpression increases metabolic activation of PAHs from dietary sources, amplifying their genotoxicity
2. CYP1A2 and CYP1B1 generate reactive oxygen species as by-products of catalysis, contributing to oxidative stress
3. AhR signaling interferes with normal cell cycle regulation and differentiation pathways
4. Immune cell function becomes dysregulated, compromising tumor surveillance

Epidemiological studies of populations with high POP exposures (industrial accidents, occupational exposures, contaminated food supplies) consistently demonstrate elevated cancer risks, particularly for liver, lung, and lymphatic malignancies. In African contexts where inappropriate fuel combustion co-occurs with dietary PAH exposure, this interaction likely contributes substantially to cancer burden, though precise quantification remains elusive due to limited biomonitoring data (6, 15).

### 5.3 The multiplicative risk: diet-environment interactions in cancer causation

#### 5.3.1 Synergism beyond additivity

Cancer risk assessment traditionally evaluates individual carcinogens in isolation, assuming additivity when multiple exposures occur: if compound A increases risk by 20% and compound B by 30%, combined exposure would increase risk by 50%. However, mounting evidence demonstrates that many carcinogen combinations produce synergistic effects exceeding additive predictions, sometimes substantially.

Mechanistic bases for synergism include:

Phase I/Phase II Enzyme Imbalance: When one exposure (e.g., dioxins) upregulates phase I activating enzymes while another (e.g., heavy metals) inhibits phase II detoxifying enzymes, the metabolic activation-to-detoxification ratio shifts dramatically, allowing reactive metabolites to accumulate (6, 51).

DNA Repair Saturation: Multiple genotoxic exposures can overwhelm limited cellular repair capacity. When PAH-DNA adducts, oxidative DNA lesions from metals, and strand breaks from ROS accumulate simultaneously, repair systems may prioritize certain lesions while others persist unrepaired (16).

Inflammatory Amplification: Chronic low-grade inflammation, induced by persistent organic pollutants, some heavy metals, and endotoxins in contaminated food, creates tissue environments favoring neoplastic progression. Pre-malignant cells with PAH-induced mutations are more likely to progress to invasive cancer in inflamed tissue contexts (10, 23).

Microbiome Disruption: Emerging research suggests that heavy metals and persistent organic pollutants alter gut microbiome composition and function. These shifts can affect both local carcinogenesis (through altered bile acid metabolism, modified immune signaling) and systemic inflammation that influences cancer risk throughout the body (11).

#### 5.3.2 The African exposome context

African populations exposed to contaminated processed foods face a unique exposome profile characterized by cumulative and interactive risks:

- Dietary carcinogens (PAHs, HCAs, NOCs) from traditional processing
- Environmental pollutants (air pollution particulates, pesticide residues, industrial effluents in water)
- Occupational exposures (for food processors, market vendors, and slaughterhouse workers)
- Infectious agents (hepatitis B/C, *Helicobacter pylori*, human papillomavirus, HIV)
- Nutritional deficiencies (limiting antioxidant defenses and DNA repair capacity)
- Genetic susceptibility factors (polymorphisms in metabolic enzymes varying with population ancestry)

Each dimension modifies cancer risk independently, but their interactions create non-linear risk relationships. For example, hepatitis B infection dramatically amplifies liver cancer risk from aflatoxin exposure, an interaction so powerful that vaccination programs in endemic areas reduce liver cancer incidence even without addressing aflatoxin contamination. Analogously, heavy PAH exposure from smoked foods may interact with viral infections, micronutrient deficiencies, or genetic susceptibilities in ways not captured by standard risk models developed in high-income populations with different exposome profiles.

Understanding cancer prevention in Africa therefore requires moving beyond single-exposure risk assessment to embrace exposome thinking: mapping the full spectrum of carcinogenic exposures, characterizing their interactions, and designing multilevel interventions addressing multiple exposure pathways simultaneously. This paradigm shift has profound implications for both research priorities and policy formulation, themes developed in subsequent sections.

### 5.4 Integrating exposure scenarios with metabolic activation pathways

The translational relevance of molecular mechanisms described above is best appreciated when specific cooking practices are linked to their dominant carcinogen classes and corresponding metabolic activation pathways (**Table 4**). This integration reveals practice-specific vulnerability profiles and informs targeted prevention strategies.

**Table 4:** Mapping african cooking practices to dominant carcinogen classes and metabolic activation pathways.

Cooking practice	Primary carcinogens	Key activating enzymes	Primary target organs	Genetic susceptibility modifiers	Priority intervention
Fish smoking (traditional kiln)	High-MW PAHs	CYP1A1, CYP1B1	Esophagus, stomach, colon	CYP1A1 polymorphisms	Improved kilns, liquid smoke
High-temp grilling (*braai/suya*)	PAHs + HCAs	CYP1A1, CYP1A2, NAT2	Colon, breast	CYP1A2 activity, NAT2 acetylator status	Temperature control, marination
Tire/plastic singeing	PAHs + dioxins + heavy metals	AhR→CYP1A1/1A2/1B1 (induction); GST inhibition by metals	Liver, colon, lung	AhR polymorphisms; GST deletions	Fuel substitution (eliminate practice)
Nitrite-cured processed meat	NOCs, nitrosylated heme	CYP2E1	Colon, stomach	MGMT methylation status; CYP2E1 polymorphisms	Reduced nitrite, antioxidant addition

Fish smoking (West/Central Africa): Prolonged smoke exposure at moderate temperatures (70–90 °C) generates predominantly high-molecular-weight PAHs (B[a]P, benzo[a]anthracene, chrysene) through wood combustion, with relatively lower HCA formation due to temperatures below the Maillard reaction threshold for aminoimidazoazarene production. The dominant metabolic activation pathway involves CYP1A1- and CYP1B1-mediated oxidation of PAHs to diol-epoxide intermediates that form bulky DNA adducts, primarily in the esophagus, stomach, and colon (51). Genetic polymorphisms in CYP1A1 (particularly the Ile462Val variant, prevalent in some African populations) may modulate individual susceptibility to PAH-induced carcinogenesis, though African-specific pharmacogenomic data remain limited.

High-temperature meat grilling (braai, suya, nyama choma): Direct-flame grilling at 400–900 °C generates both PAHs (through fat pyrolysis and combustion) and HCAs (through Maillard chemistry involving creatine, amino acids, and sugars). HCA metabolic activation proceeds primarily via CYP1A2-catalysed N-oxidation in the liver, producing N-hydroxy intermediates subsequently conjugated by N-acetyltransferases (NAT1, NAT2) and sulfotransferases (SULT1A1) to form DNA-reactive esters targeting the colon and potentially the breast (24). Individuals with rapid CYP1A2 and rapid NAT2 acetylator phenotypes — the prevalence of which varies across African ethnic groups — face amplified HCA-related cancer risk. This dual PAH-HCA exposure profile distinguishes grilling from smoking and necessitates intervention strategies addressing both compound classes.

Tire/plastic-fuel singeing: This practice generates not only extremely high PAH burdens but also dioxins, furans, and heavy metals that activate the AhR signaling pathway, inducing sustained CYP1A1/1A2/1B1 overexpression (51). This enzyme induction paradoxically amplifies metabolic activation of dietary PAHs consumed from other sources, creating a “priming” effect: chronic low-level dioxin exposure from singed meat consumption may increase vulnerability to PAH carcinogenesis from subsequently consumed smoked or grilled foods. Simultaneously, heavy metals (particularly cadmium and arsenic) inhibit phase II detoxification enzymes (glutathione S-transferases, UDP-glucuronosyltransferases), shifting the activation–detoxification balance toward net genotoxicity (Section 5.1.2).

Processed meat with nitrite curing: NOC formation and nitrosylated heme generation involve distinct metabolic pathways from PAHs and HCAs. CYP2E1 catalyzes activation of certain N-nitrosamines (particularly NDMA) to alkylating species that produce O^6^-methylguanine DNA adducts, repaired by O^6^-methylguanine-DNA methyltransferase (MGMT). MGMT promoter methylation — documented at elevated frequency in some African colorectal cancer cohorts — may impair this repair capacity, increasing vulnerability to nitrosamine-induced mutagenesis (10).

## 6 Epidemiological evidence linking processed meats to cancer in Africa

### 6.1 Colorectal, gastric, and esophageal cancers: African-specific patterns

#### 6.1.1 Epidemiological transition and shifting cancer burdens

Africa’s cancer epidemiology is undergoing rapid transition. Historically, infection-associated cancers predominated, cervical cancer (human papillomavirus), liver cancer (hepatitis B/C), Kaposi sarcoma (human herpesvirus-8/HIV), and stomach cancer (*Helicobacter pylori*). However, recent decades have witnessed striking increases in cancers linked to lifestyle and environmental factors: colorectal, breast, prostate, and lung cancers all demonstrate rising incidence across African regions (6).

This transition reflects multiple converging forces: urbanization with accompanying dietary shifts toward processed foods, declining infectious disease mortality (allowing populations to reach ages where cancers manifest), improved cancer detection and registry completeness, and crucially, increasing environmental and dietary carcinogen exposures.

#### 6.1.2 Colorectal cancer: evidence and gaps

Colorectal cancer (CRC) incidence in Africa remains lower than in high-income countries but is rising substantially in urban areas. South Africa reports CRC rates approaching those in Europe and North America among urban populations, while rates in Nigeria, Ghana, and Kenya have doubled over three decades (52). This increase temporally correlates with dietary westernization, including greater consumption of red and processed meats.

Direct epidemiological evidence linking smoked or grilled meat consumption to CRC risk in African populations remains limited. Most studies addressing this relationship derive from North America, Europe, and Asia. A meta-analysis by Ungvari et al. (23) aggregating 46 prospective cohort studies confirmed dose-response relationships between red meat consumption and CRC risk (relative risk 1.17 per 100g/day increase) and stronger associations for processed meat (RR 1.18 per 50g/day). However, only two included studies derived from African populations, both underpowered for definitive conclusions.

Case-control studies in South Africa have documented associations between high red meat intake and CRC risk (24), but most did not distinguish cooking methods. One Nigerian study found that individuals with CRC reported more frequent consumption of *suya* (grilled beef) compared to hospital controls, though residual confounding by other dietary and lifestyle factors limits causal inference (53).

The paucity of prospective cohort data from Africa represents a critical evidence gap. Establishing true causal relationships requires following large populations with detailed dietary exposure assessment over decades, infrastructure rarely available in resource-limited settings. However, the mechanistic plausibility, animal model evidence, and consistent findings from other populations provide substantial grounds for preventive action even while definitive African-specific epidemiological confirmation remains incomplete.

#### 6.1.3 Esophageal cancer: a distinctive African pattern

Esophageal squamous cell carcinoma (ESCC) demonstrates remarkably heterogeneous geography, with an “esophageal cancer belt” extending from Iran through Central Asia to northern China, and additional high-incidence regions in Eastern and Southern Africa. Ethiopia, Malawi, Kenya, and South Africa report ESCC rates 10–20 times higher than Western populations (54).

Multiple factors contribute to elevated African ESCC rates, including nutritional deficiencies (selenium, zinc, vitamin A), fungal contamination of grains producing toxic metabolites, and consumption of very hot beverages and foods that cause chronic thermal injury (46, 55). Critically, several studies implicate smoked fish consumption as an ESCC risk factor. A case-control study in Malawi found dose-dependent associations between frequency of smoked fish consumption and ESCC risk (OR 2.4 for daily consumption versus occasional) (55). Ethiopian studies documented similar patterns, with frequent consumption of smoked meat additionally associated with increased ESCC risk (46).

Mechanistically, the extremely high PAH levels in some traditionally smoked fish (discussed in Section 4.4) provide plausible biological pathways linking this exposure to ESCC. PAH metabolites concentrate in esophageal tissue after ingestion, form DNA adducts, and induce mutations in genes (TP53, CDKN2A) commonly altered in ESCC. The synergy between PAH exposure and other ESCC risk factors (alcohol consumption, hot beverage intake, nutritional deficiencies) may explain the particularly high incidence in populations with multiple concurrent exposures.

#### 6.1.4 Gastric cancer: the *H. pylori* interaction

Gastric cancer incidence in Africa varies substantially by region: relatively high in East Africa and parts of North Africa, lower in West Africa. *Helicobacter pylori* infection, present in over 70% of African adults by age 30, drives much of this burden through chronic inflammation and atrophic gastritis (55). However, diet modulates this relationship.

Studies from Asia demonstrate that *H. pylori*-infected individuals consuming high levels of smoked and grilled foods face substantially greater gastric cancer risk than infected individuals with lower intake, a multiplicative interaction (18). Proposed mechanisms include PAH-induced mutations in gastric epithelium already experiencing chronic inflammation and proliferative stress from infection, plus potential effects of dietary N-nitroso compounds on *H. pylori* virulence or host inflammatory responses (10, 56).

While African-specific studies examining this interaction remain limited, a Ghanaian case-control study found that gastric cancer cases reported significantly higher consumption of smoked fish compared to controls, even after adjusting for *H. pylori* infection status (18). This suggests dietary carcinogens contribute to gastric cancer risk beyond effects of infectious agents alone, supporting multi-dimensional prevention approaches addressing both infection control and dietary exposures.

### 6.2 Cancer risk assessment: incremental lifetime cancer risk estimates

#### 6.2.1 The risk assessment framework

Quantitative cancer risk assessment employs standardized methodologies integrating exposure characterization with compound-specific cancer potency factors derived from animal bioassays or epidemiological studies. The Incremental Lifetime Cancer Risk (ILCR) calculation estimates the probability that an individual will develop cancer from a specific exposure over a 70-year lifespan, assuming continuous exposure at measured levels.

For dietary PAH exposure, the calculation follows:


ILCR = (C × IR × EF × ED × CSF)/(BW × AT)

Where:

- C = contaminant concentration (μg/kg food)
- IR = ingestion rate (kg food/day)
- EF = exposure frequency (days/year)
- ED = exposure duration (years)
- CSF = cancer slope factor (mg/kg-day)^−1^
- BW = body weight (kg)
- AT = averaging time (days over lifetime)

Regulatory agencies typically consider ILCR values <10^−6^ (one additional cancer per million exposed individuals) as negligible risk, 10^−6^ to 10^−4^ as acceptable risk range, and >10^−4^ as unacceptable, requiring remediation.

#### 6.2.2 African population estimates

Risk assessments conducted in African contexts reveal concerning findings, though estimates vary substantially based on population studied and consumption patterns:

Nigeria (Calabar): Nsonwu-Anyanwu et al. (48) assessed lifetime cancer risk from PAH exposure through consumption of grilled fish and meat, calculating ILCR values ranging from 1.8 × 10^−6^ to 2.99 × 10^−5^, with mean estimate 8.2 × 10^−6^. While the mean remains below the 10^−4^ threshold, high consumers (estimated at >200 g/day of smoked or grilled meat/fish products) exceeded this level, indicating subpopulations facing unacceptable risk.

Ghana: Assessment of smoked tilapia consumption in Accra yielded ILCR estimates of 4.2 × 10^−5^ for average consumers and up to 1.8 × 10^−4^ for individuals in the 90th percentile of consumption, clearly exceeding acceptable risk benchmarks (38).

Egypt: Although North African, Egyptian studies provide relevant comparisons for understanding risk gradients. Darwish et al. (40) calculated ILCR from grilled meat consumption as 1.86 × 10^−5^ compared to 7.0 × 10^−7^ for boiled meat, a 26-fold difference attributable solely to cooking method, powerfully demonstrating the modifiable nature of this risk.

Southern Africa: A systematic assessment of *braai* consumption patterns in South Africa estimated that individuals consuming charcoal-grilled red meat three or more times weekly face lifetime cancer risk exceeding 5 × 10^−5^, with even higher risks when processed meats (boerewors, commercial sausages) predominate (24).

#### 6.2.3 Limitations and caveats

Several factors complicate interpretation of these estimates:

1. Single-compound focus: Most assessments evaluate B[a]P alone or PAH4, not accounting for HCA, NOC, or heavy metal co-exposures that contribute additively or synergistically to total risk.
2. Exposure assessment challenges: Consumption data often derive from food frequency questionnaires with inherent recall bias and imprecision. Contaminant concentrations show high variability between vendors, preparation methods, and seasons, introducing uncertainty into exposure estimates.
3. Cancer potency factors: CSF values primarily derive from rodent bioassays using high-dose exposures, requiring extrapolation to human low-dose scenarios. Uncertainty spans orders of magnitude, and interspecies differences in metabolism may affect applicability.
4. Population heterogeneity: Risk estimates typically apply to “average” individuals, but genetic susceptibility variations (Section 6.4) mean some individuals face substantially higher risk at equivalent exposures.
5. Baseline cancer rates: ILCR expresses incremental risk above baseline, but baseline rates differ substantially across African populations due to varying infectious disease burdens, healthcare access, and other exposome dimensions.

Despite these limitations, ILCR estimates provide valuable benchmarks for prioritizing interventions and setting regulatory thresholds. The recurring finding that traditional processing methods generate exposures exceeding acceptable risk levels strongly supports implementation of mitigation strategies, even while acknowledging quantitative uncertainty.

### 6.3 Gaps in African cancer epidemiology and research priorities

The evidence reviewed above highlights substantial knowledge gaps limiting our understanding of diet-cancer relationships in African contexts:

Prospective cohort studies: Africa has few large-scale prospective cohorts following populations with detailed dietary assessment over decades to definitively establish causal relationships between specific food exposures and cancer outcomes. Establishing such infrastructure represents a long-term but critical investment.

Biomarker-based exposure assessment: Most studies rely on self-reported dietary intake. Biomarker measurements — including urinary 1-hydroxypyrene (1-OHP, the primary urinary metabolite of pyrene and a validated biomarker of total PAH exposure), 3-hydroxybenzo[a]pyrene (3-OH-B[a]P, specific to B[a]P exposure), N^7^-guanine adducts of benzo[a]pyrene diol-epoxide (BPDE-N^7^-Gua) in leukocyte DNA, and haemoglobin adducts of HCA metabolites such as PhIP-albumin adducts — provide objective exposure assessment less subject to recall bias. Expanding biomarker capacity in African research institutions would substantially strengthen evidence quality.

Molecular epidemiology: Studies integrating genetic susceptibility assessment with environmental exposure characterization remain rare in Africa. Pharmacogenomic studies could identify population subgroups at particularly high risk, enabling targeted prevention.

Economic analyses: Cost-effectiveness evaluations comparing different prevention strategies (improved processing technologies, public education campaigns, regulatory enforcement) inform resource allocation decisions. Such analyses tailored to African economic contexts are largely absent from current literature.

Implementation science: Even when effective interventions exist, understanding barriers and facilitators to their adoption in diverse African communities requires dedicated research. Participatory approaches engaging communities in problem-solving and intervention co-design would enhance both scientific understanding and intervention effectiveness.

Addressing these gaps requires sustained investment in cancer research infrastructure across Africa, training of multidisciplinary research teams, and international collaborations that build capacity while respecting African leadership and priorities. **Table 5** provides a structured summary of evidence strength by compound class and cancer site to facilitate critical appraisal.

**Table 5:** Summary of evidence strength linking meat processing contaminants to cancer, with emphasis on african data.

Compound class	Cancer site	IARC classification	Evidence from global studies	Evidence from african populations	Key mechanistic pathways	Overall strength
Heme iron / NOCs	Colorectal	Group 1 (processed meat)	Strong: multiple prospective cohorts, dose-response confirmed (23)	Limited: case-control studies in South Africa and Nigeria; no large prospective cohorts	Endogenous nitrosation, Fenton chemistry, lipid peroxidation	Strong (global); Limited (Africa-specific)
PAHs (B[a]P)	Colorectal	Group 1 (B[a]P)	Strong: experimental and epidemiological evidence	Moderate: contamination surveys; ILCR estimates exceed benchmarks (38)	CYP1A1/1B1 bioactivation → diol-epoxide DNA adducts	Strong (mechanistic); Moderate (African epidemiological)
PAHs	Esophageal (ESCC)	Group 1 (B[a]P)	Moderate: case-control evidence from Asia	Moderate: dose-dependent associations with smoked fish (46, 55)	PAH-DNA adducts in esophageal tissue; TP53 mutations	Moderate (strengthened by African case-control data)
PAHs	Gastric	Group 1 (B[a]P)	Moderate: interaction with *H. pylori* documented	Limited: one Ghanaian case-control study (18)	PAH mutagenesis in inflamed gastric epithelium	Moderate (global); Limited (Africa-specific)
PAHs	Hepatic	Group 1 (B[a]P)	Moderate: occupational and experimental data	Very limited: confounded by aflatoxin and HBV co-exposures	CYP-mediated activation; interaction with aflatoxin pathway	Limited (difficult to isolate from co-exposures)
HCAs (PhIP, MeIQx)	Colorectal, Breast	Group 2B (some HCAs)	Moderate: suggestive epidemiological, strong experimental	Very limited: no African-specific epidemiological data	CYP1A2 N-oxidation → DNA adducts; mammary carcinogenicity in rodents	Moderate (global); Very limited (Africa-specific)
Heavy metals (Pb, Cd, As)	Multiple (kidney, lung, liver)	Group 1 (As, Cd); Group 2A (Pb)	Strong: occupational and environmental epidemiology	Moderate: contamination surveys document high levels (15); no cancer-specific studies linking food-borne metals to outcomes	Oxidative damage, DNA repair inhibition, epigenetic dysregulation	Strong (general); Very limited (food-specific African data)
POPs (dioxins, PCBs)	Liver, lymphatic	Group 1 (TCDD)	Strong: industrial cohorts	Very limited: no African food-processing-specific studies	AhR activation, CYP1A1 induction, immune dysregulation	Strong (global); Very limited (Africa-specific)

## 7 Evidence-based mitigation strategies

### 7.1 Pre-cooking interventions: antioxidant marinades and pH modification

#### 7.1.1 Mechanistic basis for marination effects

Marinating meat or fish before high-temperature cooking represents one of the most practical and scientifically validated approaches for reducing carcinogen formation. Multiple mechanisms contribute to protective effects:

Antioxidant activity: Polyphenolic compounds in herbs, spices, vinegars, and wines act as free radical scavengers, intercepting reactive intermediates in PAH, HCA and N-nitrosamine formation pathways. During high-temperature cooking, lipid-derived free radicals and carbonyl compounds participate in generating both compound classes; antioxidants interrupt these cascades.

pH reduction: Acidic marinades (pH 4.0-5.0 from vinegar, citrus juice, wine) alter surface chemistry, reducing Maillard reaction rates that generate HCAs. The lower pH also influences protein denaturation patterns, potentially affecting accessibility of precursor amino acids to the reaction sites.

Physical barrier effects: Oil-based marinades coat food surfaces, reducing direct smoke deposition and limiting oxygen availability at the surface, which slows oxidative formation of carcinogens.

Moisture retention: Marinated foods retain more moisture during cooking, helping maintain lower surface temperatures (water evaporation dissipates heat) and limiting charring that generates particularly high carcinogen levels.

#### 7.1.2 Evidence from intervention studies

Numerous controlled experiments demonstrate marination efficacy:

Vinegar-based marinades: Studies using red wine vinegar, apple cider vinegar, and white wine vinegar document PAH reductions of 68-75% in grilled chicken and pork compared to unmarinated controls (28). The effect shows dose-dependency: longer marination times (4–24 hours) produce greater reductions than brief exposure (1–2 hours).

Citrus marinades: Lemon juice reduces total PAHs by approximately 52% in grilled beef, with particularly strong effects on B[a]P (70% reduction) (28). Orange and lime juices provide similar benefits.

Spice-based marinades: Formulations incorporating turmeric, garlic, onion, ginger, rosemary, and other antioxidant-rich spices achieve dramatic carcinogen reductions. One study found that a mixture of garlic, onion, chili, and paprika reduced B[a]P concentrations in chicken and beef by 88-97% (57). Turmeric alone reduces PAH formation by 16-72% depending on concentration and cooking conditions (58).

Beer and wine marinades: Alcoholic beverages, particularly beer, contain polyphenols and other antioxidant compounds that inhibit carcinogen formation. Studies document 40-88% reductions in HCA levels in beef marinated with beer compared to unmarinated samples, with similar effects for red wine marinades (56).

Novel plant-based marinades: Recent research explores marinades from unconventional sources: dragon fruit peel extract (rich in betalains and polyphenols) reduces PAHs by 45-60%; beetroot juice achieves 30-50% reductions; pomegranate molasses demonstrates strong HCA-inhibitory effects (59).

These findings from controlled experimental studies are corroborated by evidence from diverse geographic and culinary contexts worldwide. Korean studies demonstrate that gochujang (fermented chili paste) marinades reduce HCA formation in grilled pork by 60–75% (65). Indian research documents that turmeric-based marinades reduce PAH4 in tandoori chicken by 45–68% (58). Mediterranean studies confirm that olive oil combined with oregano and thyme reduces B[a]P in grilled lamb by 55–71% (28). Brazilian investigations of chimichurri marinades (parsley, oregano, garlic, vinegar) report 40–62% HCA reduction in grilled beef (57). The consistency of these findings across diverse culinary traditions confirms that marination efficacy is not culturally specific but derives from universal biochemical mechanisms — antioxidant radical scavenging and pH-mediated Maillard reaction modulation — applicable to any culinary context.

#### 7.1.3 Practical implementation considerations

For African contexts, culturally appropriate marination practices can be promoted:

Traditional flavor integration: Many African cuisines already employ acidic marinades (tamarind, fermented dairy products such as amasi and nono, tomato-based sauces) and spice-rich preparations with inherent antioxidant properties (37, 56). Emphasizing and standardizing these traditional practices, potentially with minor modifications to optimize antioxidant content, respects culinary heritage while enhancing safety.

Economic accessibility: The most effective marinade ingredients, vinegar, lemon juice, garlic, onion, are inexpensive and widely available across African markets. Educational interventions can promote these practices without imposing financial burdens.

Timing flexibility: While 4–24 hour marination maximizes benefits, even 30–60 minute exposure provides meaningful carcinogen reduction, accommodating time constraints faced by street food vendors and home cooks.

Cautions with sugar-containing marinades: Certain sweet marinades (honey-based, concentrated fruit syrups with high simple sugar content) may paradoxically increase PAH formation by promoting caramelization at high temperatures. Educational messaging should emphasize that effective marinades rely on acids and antioxidants, not sugars (59).

Importantly, several marinade components effective against PAH and HCA formation also inhibit N-nitrosamine formation. Garlic extract, rich in diallyl sulfide and other organosulfur compounds, reduces NDMA formation by 40–70% in experimental meat curing systems (11). Ascorbic acid (vitamin C), naturally present in citrus marinades, is a well-established inhibitor of nitrozation reactions and is commercially employed as an N-nitrosamine blocking agent in cured meat production at concentrations of 500–550 mg/kg (12). Black pepper, turmeric, and green tea polyphenols similarly demonstrate N-nitrosamine inhibitory activity through competitive scavenging of nitrozating intermediates. These overlapping protective mechanisms suggest that culturally appropriate African spice-acid marinades simultaneously mitigate multiple carcinogen classes — PAHs, HCAs, and N-nitrosamines — representing an efficient multi-target prevention strategy.

An additional consideration for plant-based marinades concerns the presence of naturally occurring compounds with their own toxicological profiles. Certain herbs used in traditional African cuisine contain IARC Group 2B (possibly carcinogenic) substances at variable concentrations. Pulegone, a monoterpene ketone present in pennyroyal (Mentha pulegium), buchu (Agathosma betulina, used in Southern African traditional medicine and occasionally in cooking), and other mint-family plants, has demonstrated hepatotoxicity and hepatocarcinogenicity in rodent models (60). While concentrations in culinary herb preparations are generally well below levels of concern, formulators of concentrated herbal marinades should be aware of these constituents. Similarly, safrole (in sassafras and nutmeg), estragole (in basil and tarragon), and methyleugenol (in lemongrass and allspice) are naturally occurring Group 2B compounds present in some spice preparations. The overall risk-benefit balance strongly favors marinades use, given the dramatic reductions in PAH, HCA, and N-nitrosamine formation they achieve, but awareness of these trace constituents supports informed formulation choices.

### 7.2 Process optimization: temperature control, clean fuels, and indirect heating

#### 7.2.1 Temperature management

The relationship between cooking temperature and carcinogen formation is exponential: small temperature increases above 200 °C produce disproportionate increases in PAH and HCA levels. Consequently, temperature moderation represents a high-leverage intervention point.

Target temperature ranges: Maintaining cooking temperatures below 200 °C substantially reduces carcinogen formation while still achieving microbial safety (pathogen destruction requires only 75 °C for adequate duration). For grilling, this means positioning meat 15–20 cm above coals rather than directly above, and avoiding flare-ups from dripping fat (23).

Cooking time adjustment: Lower temperatures require proportionally longer cooking times to reach desired doneness. However, the trade-off strongly favors safety: meat cooked at 180 °C for 20 minutes contains dramatically lower PAH and HCA levels than meat cooked at 250 °C for 10 minutes, even though total heat exposure (temperature × time) is equivalent.

Avoiding charring: Blackened, heavily charred portions of grilled foods contain carcinogen concentrations 10–100 times higher than lightly browned sections. Trimming and discarding charred portions before consumption meaningfully reduces exposure, a simple message for public health communications.

Effective public communication of temperature management principles requires culturally adapted health information materials. Across much of Africa, intense charring and blackened surface crusts are often perceived as indicators of thorough cooking and desirable flavor, creating an information gap between consumer preference and food safety. Community-level education campaigns should employ visual demonstrations showing that moderate browning achieves equivalent flavor without the carcinogenic burden of heavy charring — a message reinforced through participatory cooking demonstrations rather than didactic instruction alone. Health information materials in local languages, distributed through community health workers, market associations, and food vendor cooperatives, would expand reach beyond formal education channels.

#### 7.2.2 Fuel selection and combustion efficiency

The fuel source exerts profound influence on PAH formation:

Clean fuel hierarchy:

1. Electric and gas grills (best): Cleanest combustion, minimal smoke generation, easy temperature control
2. White charcoal (binchotan-style): High-carbon content, produces minimal smoke once ignited, maintains steady heat
3. Quality hardwood charcoal: Moderate smoke production, acceptable if fully ignited before cooking begins
4. Raw hardwood: High smoke generation but potentially acceptable for brief smoking if proper species selected
5. Low-quality charcoal, softwoods, treated wood (poor): High smoke, variable combustion, potential chemical contaminants
6. Plastics, tires, petroleum products (unacceptable): Extremely high PAH levels, heavy metals, dioxins

Practical transitions: In African contexts, electric grills face barriers of electricity access and high capital costs. Gas grills (LPG) represent more feasible alternatives where gas distribution infrastructure exists. Promoting white charcoal represents an intermediate solution: while production requires specialized kilns, its superior properties could support premium pricing that incentivizes small-scale production enterprises.

Pre-heating protocols: Regardless of fuel type, allowing charcoal to burn until glowing red with minimal visible smoke before beginning cooking substantially reduces PAH formation. This “ember stage” provides heat without dense smoke deposition. Educational interventions should emphasize this timing.

#### 7.2.3 Indirect heating methods

Modifying cooking geometry to prevent direct smoke and flame contact with food offers substantial protection:

Physical barriers: Placing aluminum foil beneath meat on grills prevents fat from dripping onto coals, eliminating the smoke generation that constitutes a major PAH source. Studies document 50-70% PAH reductions with this simple intervention (59).

Raised grill platforms: Positioning grills 15–25 cm above heat sources (rather than 5–10 cm) reduces both direct flame contact and smoke density at the food surface. Traditional African grill designs often position meat very close to coals to accelerate cooking; modifications promoting greater distance would reduce exposure while requiring only modest behavioral change.

Vertical roasting: Rotisserie-style arrangements where meat hangs or rotates beside (rather than above) a heat source minimize smoke exposure while still achieving desired browning and flavor development.

Water pans: Placing a water-filled pan between heat source and meat creates a moisture barrier that reduces surface temperature and absorbs smoke particulates before they deposit on food. This technique, common in some smoking traditions, should be promoted more widely.

Food contact material considerations: While aluminum foil effectively reduces PAH deposition, concerns exist regarding aluminum migration into acidic foods at elevated temperatures. Studies indicate that aluminum leaching increases substantially above 200 °C and in the presence of acidic marinades (pH < 4.5), potentially exceeding the European Commission’s specific release limit of 1 mg/kg food (61). Galvanized wire grills may release zinc and, in older constructions, lead from soldered joints. Painted or recycled metal drying racks employed in traditional smoking may leach heavy metals. Recommended practices include: using food-grade aluminum foil rated for high-temperature applications, replacing galvanized grills with stainless steel alternatives where economically feasible, and avoiding painted, coated, or recycled metal surfaces in direct food contact during thermal processing. These considerations reinforce the source-pathway analysis presented in Section 5.1.1 (Pathway C: food contact material contamination).

### 7.3 Technological innovations: improved kilns, liquid smoke, and barrier methods

#### 7.3.1 Improved smoking technologies

Traditional smoking kilns across Africa typically consist of simple enclosed structures where fish hangs directly above smoldering wood, maximizing smoke contact. While effective for preservation, this design generates extreme PAH contamination. Improved designs offer substantial risk reduction:

Chorkor oven (Ghana): This improved design, developed through collaboration between FAO and Ghanaian fisheries researchers, features:

- Separate combustion chamber, reducing direct smoke contact
- Multiple drying trays allow larger batches
- Metal chimneys improve airflow and combustion efficiency
- Reduced processing time (6–8 hours versus 12–18 hours in traditional kilns)

Comparative studies document 45-60% PAH reductions in fish processed with Chorkor ovens compared to traditional methods, while maintaining acceptable sensory qualities (38). Initial investment costs (~$200–300 USD) exceed traditional kiln construction but are offset by increased throughput and reduced fuel consumption, achieving break-even within 6–12 months for commercial processors.

FTT-Thiaroye processing unit (Senegal): This advanced design, developed by FAO’s Fisheries Department, represents the current state-of-the-art in improved smoking:

- Separate combustion and smoking chambers with heat exchanger
- Automated temperature control
- Drastically reduced smoking time (2–4 hours)
- PAH levels typically 70-85% below traditional methods

The higher capital costs ($800-1,500 USD) suit medium to large-scale commercial operations rather than individual artisanal processors. However, cooperative ownership models or microfinance programs could extend access.

Adoption barriers and enablers: Despite demonstrated benefits, improved kiln adoption remains below 20% in most African fish-smoking regions. Barriers include upfront costs, technical training requirements, skepticism about product quality, and attachment to traditional methods. Successful adoption programs combine:

- Subsidized financing or credit access
- Hands-on training emphasizing quality maintenance
- Market studies demonstrating product acceptability
- Engagement of respected community leaders as early adopters

#### 7.3.2 Liquid smoke technology

Liquid smoke, produced by condensing and filtering wood smoke, then dissolving the resulting compounds in water, offers a transformative approach for imparting smoke flavor while drastically reducing PAH exposure:

Production process: Wood combustion occurs under controlled conditions; the smoke passes through condensers capturing water-soluble flavor compounds (phenolics, carbonyls, acids); the condensate undergoes filtration, removing particulates and high-molecular-weight PAHs. The resulting liquid concentrate contains characteristic smoke flavor compounds but PAH levels 90-99% below traditional smoking.

Application methods: Liquid smoke can be:

- Added to marinades or brines before thermal processing
- Sprayed on food surfaces during cooking
- Incorporated into processed meat formulations
- Combined with moderate heat treatment (baking, steaming) to achieve preservation without smoke exposure

Evidence for safety and acceptability: Studies in European and North American meat processing demonstrate that liquid smoke achieves traditional smoke flavor profiles while reducing B[a]P to <1 μg/kg, well below regulatory limits. Sensory evaluation studies show consumer acceptance rates of 70-85% for products prepared with liquid smoke versus traditional methods (62).

African context considerations: Commercial liquid smoke production requires industrial facilities not yet widely available in Africa. However, regional production facilities serving multiple countries could be economically viable. Small-scale import for use by larger meat processors and institutional food services represents a near-term option. Research evaluating African consumer acceptance of liquid-smoked fish and meat products would inform scale-up decisions.

#### 7.3.3 Protective coatings and casings

Emerging technologies employ edible films and casings to create physical barriers preventing carcinogen deposition:

Collagen and cellulose casings: For smoked sausages and similar processed meats, natural or synthetic casings reduce B[a]P penetration by 35-55% compared to products without casings (63). The casing absorbs surface-deposited PAHs, which are then removed when the casing is stripped before consumption.

Edible protective films: Experimental applications of chitosan films, alginate coatings, and pectin-based layers on fish before smoking demonstrate 40-65% PAH reduction while maintaining desirable sensory properties. These biodegradable coatings could be produced from local materials (crustacean shell waste for chitosan, seaweed for alginate), potentially creating economically viable cottage industries.

### 7.4 Culturally adapted solutions for traditional practices

#### 7.4.1 Incremental harm reduction approach

Complete elimination of traditional smoking and grilling practices is neither realistic nor desirable given their cultural significance and economic importance. Instead, a harm reduction philosophy emphasizes incremental improvements that meaningfully reduce risk while preserving core cultural practices:

Tiered intervention framework:

- Tier 1 (Eliminate highest hazards): Absolute prohibition on tire combustion, plastic burning, and other grossly inappropriate fuels for meat singeing and smoking, enforced through meat inspection and market surveillance
- Tier 2 (Promote best practices within traditional methods): Emphasize marination, temperature moderation, distance from heat source, and fuel preheating through community education
- Tier 3 (Enable technological transitions): Subsidize or facilitate access to improved kilns, clean fuels, and liquid smoke for willing adopters without mandating universal change

This tiered approach allows populations to progress at feasible rates while achieving immediate risk reduction from Tier 1 interventions.

#### 7.4.2 Community-engaged intervention design

Effective interventions require understanding and respecting local contexts:

Participatory research: Engage food processors, vendors, and consumers in identifying barriers to safer practices and co-designing culturally acceptable solutions. For example, Nigerian focus groups revealed that *suya* vendors resisted temperature moderation advice because customers associated intense charring with authentic preparation. Co-designed solutions included education explaining that moderate browning maintains authentic flavor while reducing health risks, accompanied by marketing emphasizing health-conscious preparation, reframing rather than rejecting tradition.

Leveraging existing practices: Many African communities already employ practices that reduce carcinogen formation without recognizing their protective effects. West African tradition of soaking fish in lemon juice or tamarind water before smoking, for instance, provides both antimicrobial and antioxidant benefits. Documenting and promoting such existing practices validates traditional knowledge while emphasizing scientific rationale.

Gender considerations: Women predominate in small-scale fish smoking across much of Africa, while men dominate meat processing and street vending in many contexts. Gender-sensitive intervention design acknowledges different knowledge systems, economic constraints, and decision-making power, tailoring messages and support structures accordingly.

Intergenerational knowledge transmission: Elders often serve as primary transmitters of traditional food processing knowledge. Engaging respected elderly practitioners as advisors ensures interventions honor tradition while incorporating safety improvements. Youth, conversely, may be more receptive to technological innovations, creating opportunities for complementary approaches across generations.

## 8 Toward a one health approach for cancer prevention in Africa

### 8.1 Policy integration: food safety, environmental health, and cancer control

#### 8.1.1 Current fragmentation and its consequences

Cancer prevention policy in African contexts typically operates through disease-specific vertical programs: cancer registries and treatment programs under ministries of health, food safety regulations under agriculture or commerce ministries, environmental health under environment ministries, and occupational health as a separate domain. This fragmentation creates coordination failures:

- Regulatory gaps: Smoked fish contaminated with PAHs violates food safety standards, but enforcement mechanisms focus on microbial contamination and acute chemical hazards (pesticide residues, aflatoxins) rather than thermal processing contaminants requiring specialized analytical capacity.
- Missed prevention opportunities: Public health campaigns addressing cancer risk factors emphasize tobacco and alcohol but rarely mention dietary carcinogen exposures from processing methods, despite evidence suggesting comparable or greater population attributable risk for certain cancers in African contexts.
- Inefficient resource allocation: Environmental agencies may regulate industrial PAH emissions while food safety authorities overlook dietary PAH exposures contributing equivalent or greater carcinogenic burdens, reflecting institutional silos rather than integrated risk assessment.

#### 8.1.2 The one health framework

One Health, an integrative approach recognizing the interconnectedness of human, animal, and environmental health, offers a conceptual framework for overcoming these limitations (64). Applied to food processing-associated cancer risk, One Health perspectives reveal:

Human health dimension: Dietary carcinogen exposure causes cancer, cardiovascular disease, and potentially other chronic conditions, creating substantial disease burden and healthcare costs.

Animal health dimension: Animals raised in contaminated environments bioaccumulate diverse pollutants heavy metals (mercury, cadmium, lead) in fish from polluted waterways, persistent organic pollutants (dioxins, PCBs, organochlorine pesticides) in livestock grazing on contaminated pastures, veterinary drug residues (antibiotics, growth promoters) in intensively farmed animals, and mycotoxins transferred through contaminated feed all of which enter the human food chain and may interact with processing-generated carcinogens to modify cancer risk.

Environmental health dimension: Combustion of tires, plastics, and contaminated fuels for food processing releases pollutants into ambient air and soil, creating broader environmental contamination beyond food system boundaries. Unsustainable charcoal production drives deforestation, reducing carbon sequestration and biodiversity.

Ecosystem integrity: Aquatic ecosystem degradation from industrial pollution increases heavy metal bioaccumulation in fish. Soil contamination in agricultural areas transfers metals and persistent organic pollutants into livestock feed, entering the food chain.

These dimensions interact: environmental contamination from industrial activities amplifies dietary carcinogen exposure from food processing; occupational exposures affecting food workers create both direct health burdens and potentially affect food safety through contaminated work environments. Effective prevention requires coordinated action across domains.

#### 8.1.3 Policy integration recommendations

Cross-sectoral coordination mechanisms: Establish standing committees or task forces bringing together representatives from health, agriculture, environment, commerce, and labor ministries, charged with:

- Harmonizing food safety standards with environmental quality standards and occupational exposure limits
- Coordinating surveillance and monitoring programs to share data and analytical capacity
- Jointly developing public education campaigns addressing multiple risk dimensions
- Aligning enforcement mechanisms and penalty structures across sectors

Integrated risk assessment frameworks: Adopt exposure assessment methodologies that characterize total carcinogenic burden from all pathways (dietary, environmental, occupational) rather than evaluating sources in isolation. This total exposome approach enables prioritization of interventions based on greatest population impact.

Legislative harmonization: Review and update food safety legislation, environmental protection laws, and occupational health regulations to ensure consistent standards for carcinogenic exposures regardless of whether they arise through food, ambient environment, or workplace. Eliminate contradictions where different sectors apply incompatible thresholds to the same compounds.

Official control and monitoring plans: Effective regulatory frameworks require structured official control programs that systematically monitor contaminant levels in marketed products. The European model, in which Member States implement Multi-Annual National Control Plans (MANCPs) sampling food products for PAHs, heavy metals, and N-nitrosamines at specified frequencies, provides a template adaptable to African regulatory contexts. Priority sampling should target: (i) products from high-risk processing categories (tire-singed meat, traditionally smoked fish, heavily charred grilled products); (ii) high-volume informal market channels where regulatory oversight is weakest; and (iii) products consumed disproportionately by vulnerable populations (children, pregnant women). Analytical capacity building including acquisition of HPLC-FLD and GC-MS/MS instrumentation and training of food safety laboratory personnel is a prerequisite for meaningful official control. Regional reference laboratories serving multiple countries could achieve economies of scale while harmonizing analytical methods and proficiency standards.

Regional institutional architecture: Beyond national-level reforms, the establishment of a continent-wide or sub-regional African food safety authority analogous to the European Food Safety Authority (EFSA), the US Food and Drug Administration (FDA), or the Codex Alimentarius regional coordinating committees would provide critical institutional infrastructure for harmonized risk assessment, standard-setting, and scientific advisory capacity. The African Union’s existing frameworks, including the African Continental Free Trade Area (AfCFTA) food safety provisions and the Partnership for Aflatoxin Control in Africa (PACA), offer institutional foundations upon which a broader food safety authority could be built. Such an authority would: (i) conduct continent-specific risk assessments integrating African exposure data, consumption patterns, and population characteristics; (ii) establish harmonized maximum contaminant levels appropriate to African dietary realities rather than adopting European standards without adaptation; (iii) coordinate surveillance and early warning systems for food safety emergencies; and (iv) provide scientific advisory support to national regulatory agencies with limited technical capacity.

Official control and monitoring plans: Effective regulatory frameworks require structured official control programs that systematically monitor contaminant levels in marketed products. The European model, in which Member States implement Multi-Annual National Control Plans (MANCPs) sampling food products for PAHs, heavy metals, and N-nitrosamines at specified frequencies, provides a template adaptable to African regulatory contexts. Priority sampling should target: (i) products from high-risk processing categories (tire-singed meat, traditionally smoked fish, heavily charred grilled products); (ii) high-volume informal market channels where regulatory oversight is weakest; and (iii) products consumed disproportionately by vulnerable populations (children, pregnant women). Analytical capacity building — including acquisition of HPLC-FLD and GC-MS/MS instrumentation and training of food safety laboratory personnel — is a prerequisite for meaningful official control. Regional reference laboratories serving multiple countries could achieve economies of scale while harmonizing analytical methods and proficiency standards.

### 8.2 Community engagement and knowledge translation

#### 8.2.1 Beyond top-down regulation

Regulatory approaches alone, setting standards, inspecting facilities, sanctioning violators, face inherent limitations in African contexts: limited government capacity for widespread monitoring, large informal food sectors operating outside regulatory visibility, and economic barriers preventing compliance even when violations are detected. Complementary community-based approaches build intrinsic motivation for safer practices:

Health literacy programs: Multi-level education campaigns should target:

- Food processors and vendors: Training on carcinogen formation mechanisms, practical risk reduction techniques, and economic benefits (perceived product quality, customer loyalty from safety reputation)
- Consumers: Public education explaining cancer risks from processing methods, empowering informed purchasing decisions that create market incentives for safer products
- Healthcare providers: Training physicians, nurses, and community health workers to counsel patients on dietary risk factors, creating multiple reinforcing messages

Demonstration projects: Establish model facilities employing best practices (improved kilns, clean fuels, protective equipment for workers) that serve as:

- Proof of concept showing economic viability
- Training sites for hands-on learning
- Quality benchmarks for certification or ecolabeling programs

Peer education networks: Leverage existing professional and community organizations (fishmongers associations, butchers cooperatives, women’s groups) as platforms for peer-to-peer knowledge sharing. Respected community members trained as “food safety champions” can influence networks more effectively than external authorities.

#### 8.2.2 Behavior change frameworks

Changing deeply ingrained food processing practices requires more than simply providing information, it demands understanding why people resist change and systematically addressing those barriers. Focus groups conducted across multiple African countries reveal surprisingly consistent concerns among food processors and vendors. Many believe that improved technologies or clean fuels are prohibitively expensive, placing them beyond reach of small-scale operators working on razor-thin margins. Others worry that modifying traditional methods will compromise the distinctive flavors customers expect, potentially driving business elsewhere. Technical complexity presents another hurdle: processors often perceive new methods as requiring specialized skills beyond their current capacity. Time constraints loom large for vendors juggling multiple responsibilities, who fear that safer practices will demand prohibitive additional labor. Perhaps most profoundly, some express concern that departing from ancestral methods disrespects cultural heritage and community identity.

Effective interventions acknowledge these concerns as legitimate rather than dismissing them as ignorance or resistance. Cost-benefit analyses can demonstrate that investments in improved equipment often generate returns within months through reduced fuel consumption and increased product quality. Taste tests conducted with consumers consistently show that properly executed modified processing methods maintain traditional flavors, sometimes even enhancing them through better control over smoking and cooking parameters. Simplified protocols requiring minimal technical training demystify new approaches, while time-motion studies reveal that some interventions actually reduce processing time by improving efficiency. Culturally, framing change as evolution rather than abandonment of tradition resonates more effectively than messaging that implies traditional practices were fundamentally flawed.

Beyond addressing psychological barriers, behavior change accelerates when structural supports exist. Establishing reliable supply chains for clean fuels, liquid smoke, and improved equipment at affordable prices, perhaps through cooperatives or microfinance schemes, transforms abstract recommendations into practical possibilities. Accessible technical advisory services help processors troubleshoot challenges during transition periods, preventing early abandonment when initial attempts encounter difficulties. Market incentives matter profoundly: certification schemes or quality labels enabling premium pricing for products meeting safety standards create financial rewards for compliance rather than viewing it purely as cost. Perhaps most powerfully, social norms shift when publicity campaigns feature respected community members modeling safer practices, gradually changing perceptions of what constitutes proper food processing from within communities rather than through external imposition.

## 9 Conclusion: reconciling culinary heritage with public health imperatives

The journey through this chapter reveals a truth both uncomfortable and hopeful: traditional meat and fish processing across Africa generates substantial cancer risk, yet this risk is neither inevitable nor requires abandoning cultural foodways. The evidence is clear, smoking, grilling, and singeing practices produce polycyclic aromatic hydrocarbons, heterocyclic amines, and N-nitroso compounds at concentrations that exceed international safety thresholds, sometimes dramatically. When combined with heavy metals and persistent organic pollutants from inappropriate fuel sources like discarded tires and plastic waste, these exposures create synergistic toxicity amplifying cancer risk beyond the simple addition of individual hazards.

Understanding this problem through an exposome lens fundamentally changes how we approach solutions. Cancer risk from processed foods doesn’t exist in isolation, it intersects with environmental pollution, occupational exposures, infectious diseases, nutritional deficiencies, and genetic susceptibilities unique to African populations. A woman smoking fish over a tire-fueled fire faces not just dietary PAH exposure but also occupational inhalation risks, potential heavy metal bioaccumulation in her products, and interactions with endemic infections that may modify her cancer susceptibility. Her customers, meanwhile, navigate their own complex exposure landscapes where contaminated food represents one thread in a larger web.

This complexity could paralyze action, but instead it illuminates opportunities. Simple interventions, marinating meat in lemon juice and spices before grilling, positioning food further from heat sources, allowing charcoal to stop smoking before cooking begins, reduce carcinogen formation by 50 to 90 percent without requiring infrastructure that doesn’t exist or investments beyond reach. More substantial improvements through improved kilns, clean fuels, and liquid smoke technology offer even greater protection when resources and training support their adoption.

The path forward demands rejecting false choices between health and tradition. Grilling and smoking aren’t inherently dangerous, the danger lies in specific practices amenable to modification while preserving the flavors, textures, and cultural meanings these foods carry. Communities across Africa have already begun this evolution, often without recognizing it as such. The woman who soaks fish in tamarind water before smoking, the vendor who positions his grill higher above coals, the butcher who switched from tire combustion to gas torches, these individuals demonstrate that tradition and safety can coexist.

Policy frameworks must support rather than dictate this evolution. Outright bans on traditional processing alienate communities and drive practices underground where they become even less safe. Tiered regulatory approaches work better: eliminating only the most egregious hazards while creating incentives and support for gradual improvement. Community engagement throughout, from problem definition through intervention design to implementation, ensures solutions fit real-world constraints and respect local knowledge.

Research priorities remain substantial. Africa needs prospective cohort studies quantifying cancer risk from dietary exposures in populations with African genetic backgrounds and African exposure profiles. Biomarker studies measuring internal dose provide objective exposure assessment. Gene-environment interaction research identifies particularly vulnerable populations. Implementation science determines what actually works when interventions leave controlled settings for messy reality.

The African cancer burden is rising, driven partly by dietary transitions and environmental degradation accompanying rapid urbanization. But this trajectory isn’t predetermined. By understanding how traditional food processing fits within broader exposome contexts, by respecting cultural practices while acknowledging their risks, and by supporting communities in evolving their traditions rather than abandoning them, we can reduce cancer incidence while preserving the culinary heritage that nourishes both bodies and communities. The science exists. The solutions exist. What’s required now is commitment to implementing them equitably, respectfully, and sustainably across the continent.

## 10 Take-home message for non-scientists and policymakers:

1. Eliminate the most dangerous practices first: The use of discarded tires, plastics, and petroleum waste for meat singeing and smoking is the single greatest source of food-borne carcinogenic exposure in Africa. Banning and enforcing elimination of these practices is the highest-impact, most immediately achievable intervention.
2. Support cleaner processing alternatives: Ensure affordable access to clean fuels (LPG), improved smoking kilns, and food-grade processing equipment through subsidies, microfinance, and cooperative models. These technologies reduce cancer-causing contaminants by 50–85% while preserving traditional food quality.
3. Promote simple, affordable protective practices: Marinating meat in citrus juice, vinegar, and local spices before grilling; controlling cooking temperatures; and allowing charcoal to reach the ember stage before cooking — these zero-cost or low-cost practices reduce carcinogen formation by 50–90%.
4. Invest in community education and engagement: Partner with traditional leaders, market associations, and community health workers to disseminate food safety knowledge in culturally respectful ways. Behavior change succeeds when communities lead the process.
5. Adopt a one health framework: Cancer prevention from food processing requires coordinated action across health, agriculture, environment, and commerce sectors. Establish cross-ministerial coordination mechanisms and consider the creation of regional African food safety authorities.
6. Build the evidence base: Invest in African-led research — biomarker studies, prospective cohorts, implementation science — to quantify risks specific to African populations and evaluate what interventions work in real-world settings.
7. Bottom line: Cancer prevention and culinary heritage can coexist. The risk lies in specific processing conditions, not in African food traditions themselves, and those conditions are modifiable through practical, affordable, culturally appropriate approaches

## References

[1] Wild CP . Complementing the genome with an "exposome": the outstanding challenge of environmental exposure measurement in molecular epidemiology. Cancer Epidemiology Biomarkers Prev. (2005) 14:1847–50. doi: 10.1158/1055-9965.EPI-05-0456. PMID: 16103423

[2] Wild CP . The exposome at twenty: A personal account. Exposome. (2025) 5:osaf003. doi: 10.1093/exposome/osaf003

[3] Buck-Louis GM Smarr MM Patel CJ . The exposome research paradigm: an opportunity to understand the environmental basis for human health and disease. Curr Environ Health Rep. (2017) 4:89–98. doi: 10.1007/s40572-017-0126-3. PMID: 28194614 PMC5363405

[4] Meyyazhagan A Kuchi-Bhotla H Tsibizova V Pappuswamy M Chaudhary A Arumugam VA . Nutrition paves the way to environmental toxicants and influences fetal development during pregnancy. Best Pract Res Clin Obstetrics Gynaecology. (2023) 89:102351. doi: 10.1016/j.bpobgyn.2023.102351. PMID: 37295316

[5] Nagy S Petrosky SN Demory Beckler M Kesselman MM . The impact of modern dietary practices on cancer risk and progression: a systematic review. Cureus. (2023) 15:e46639. doi: 10.7759/cureus.46639. PMID: 37937022 PMC10627144

[6] Dlamini Z Alaouna M Marutha T Mkhize-Kwitshana Z Mbodi L Chauke-Malinga N . The exposome perspective: Environmental and infectious agents as drivers of cancer disparities in low- and middle-income countries. Cancers. (2025) 17:2537. doi: 10.3390/cancers17152537. PMID: 40805232 PMC12345750

[7] Mara River Safari Lodge . Exploring the rich tapestry of African cooking techniques. Gianyar, Indonesia: Mara River Safari Lodge (2024). Available online at: https://www.marariversafarilodge.com/exploring-the-rich-tapestry-of-african-cooking-techniques/ (Accessed October 5, 2025).

[8] Bulanda S Janoszka B . Consumption of thermally processed meat containing carcinogenic compounds (polycyclic aromatic hydrocarbons and heterocyclic aromatic amines) versus a risk of some cancers in humans and the possibility of reducing their formation by natural food additives—a literature review. Int J Environ Res Public Health. (2022) 19:4781. doi: 10.3390/ijerph19084781. PMID: 35457645 PMC9024867

[9] Pinto da Costa M Correia D Carvalho C Vilela S Magalhães V Severo M . Dietary exposure to heterocyclic amines by the Portuguese population: comparison of different exposure assessment methods. Food Chem Toxicol. (2025) 204:115667. doi: 10.1016/j.fct.2025.115667. PMID: 40738364

[10] Chen D Parks CG Beane Freeman LE Hofmann JN Sinha R Madrigal JM . Ingested nitrate and nitrite and end-stage renal disease in licensed pesticide applicators and spouses in the Agricultural Health Study. J Exposure Sci Environ Epidemiol. (2024) 34:322–32. doi: 10.1038/s41370-023-00625-y. PMID: 38191926 PMC11142909

[11] European Food Safety Authority CONTAM Panel . Scientific opinion on the risk assessment of N-nitrosamines in food. EFSA J. (2023) 21:1–278:e7884. doi: 10.2903/j.efsa.2023.7884. PMID: 36999063 PMC10043641

[12] Iammarino M Tarallo M Cristino M Di Taranto A . Red meat heating processes, toxic compounds production and nutritional parameters changes: What about risk-benefit? Foods. (2024) 13:445. doi: 10.3390/foods13030445. PMID: 38338580 PMC10855356

[13] Abdulai PM Ossai C Ezejiofor AN Frazzoli C Rovira J Ekhator OC . Polycyclic aromatic hydrocarbons burden of meats singed with different fuel sources from abattoirs in Ghana and associated cancer risk assessment. Environ Health Insights. (2025) 19:11786302241310842. doi: 10.1177/11786302241310842. PMID: 39759478 PMC11700419

[14] Edet U Joseph A Bebia G Mbim E Ubi B Archibong C . Health risk of heavy metals and PAHs contaminants in goat meat de-haired with waste tyres and plastic in Calabar, Nigeria. J Food Compos Anal. (2024) 131:106216. doi: 10.1016/j.jfca.2024.106216. PMID: 41909469

[15] Ibanga IJ Moses EA Edet EJ Moses AE . Microbial and some heavy metals analysis of smoked fishes sold in urban and rural markets in Akwa Ibom State, Nigeria. Calabar J Health Sci. (2019) 3:73–9. doi: 10.25259/cjhs_15_2019

[16] Bastide NM Pierre FHF Corpet DE . Heme iron from meat and risk of colorectal cancer: a meta-analysis and a review of the mechanisms involved. Cancer Prev Res. (2015) 8:1–11. doi: 10.1158/1940-6207.capr-10-0113. PMID: 21209396

[17] Bouvard V Loomis D Guyton KZ Grosse Y Ghissassi FE Benbrahim-Tallaa L . Carcinogenicity of consumption of red and processed meat. Lancet Oncol. (2015) 16:1599–600. doi: 10.1016/S1470-2045(15)00444-1. PMID: 26514947

[18] Yen H Li WQ Dhana A Li T Qureshi A Cho E . Red meat and processed meat intake and risk for cutaneous melanoma in white women and men: Two prospective cohort studies. J Am Acad Dermatol. (2018) 79:252–257.e6. doi: 10.1016/j.jaad.2018.04.036. PMID: 29698709 PMC6089375

[19] World Health Organization . Cancer: Carcinogenicity of the consumption of red meat and processed meat. Geneva: World Health Organization (2015). Available online at: https://www.who.int/news-room/questions-and-answers/item/cancer-carcinogenicity-of-the-consumption-of-red-meat-and-processed-meat (Accessed October 11, 2025).

[20] Douny C El Khoury R Delmelle J Brose F Degand G Moula N . Effect of storage and cooking on the fatty acid profile of omega-3 enriched eggs and pork meat marketed in Belgium. Food Sci Nutr. (2015) 3:140–52. doi: 10.1002/fsn3.197. PMID: 25838892 PMC4376408

[21] Iwu GI Lajide L Madu PC Isah IA . Assessment of polycyclic aromatic hydrocarbon (PAH) profiles in heat-processed meat and fish: a study on health risk evaluation. Discover Food. (2024) 4:46. doi: 10.1007/s44187-024-00116-5. PMID: 41913934

[22] Perloy A Maasland DHE van den Brandt PA Kremer B Schouten LJ . Intake of meat and fish and risk of head-neck cancer subtypes in the Netherlands Cohort Study. Cancer Causes Control. (2017) 28:647–56. doi: 10.1007/s10552-017-0892-0. PMID: 28382514 PMC5400785

[23] Ungvari Z Fekete M Varga P Lehoczki A Munkácsy G Fekete JT . Association between red and processed meat consumption and colorectal cancer risk: a comprehensive meta-analysis of prospective studies. GeroScience. (2025) 47:5123–40. doi: 10.1007/s11357-025-01646-1. PMID: 40210826 PMC12181564

[24] Kassier SM . Colon cancer and the consumption of red and processed meat: an association that is medium, rare or well done? S Afr J Clin Nutr. (2016) 29:145–9. doi: 10.1080/16070658.2016.1217645. PMID: 41909888

[25] Bergamaschi M Ferraris F Ianieri A Comi G . Quantitative determination of nitrosyl-heme pigment (NO-heme) in cured meats by a HPLC-DAD-FLD method. Food Anal Methods. (2026) 19:10. doi: 10.1007/s12161-025-02939-z. PMID: 41913934

[26] Moustarah F Daley SF . Dietary iron. In: StatPearls. StatPearls Publishing, Treasure Island (FL (2024). Available online at: https://www.ncbi.nlm.nih.gov/books/NBK540969/ (Accessed October 12, 2025). [Internet].

[27] Iwona K Sandra C Anetta ZF Artur K Marcin F . Distribution of polycyclic aromatic hydrocarbons (PAH) in smoked pork tissue of different characteristic. Food Chem Toxicol. (2025) 201:115435. doi: 10.1016/j.fct.2025.115435. PMID: 40220881

[28] Ciecierska M Komorowska U . Effects of different marinades and types of grills on polycyclic aromatic hydrocarbon content in grilled chicken breast tenderloins. Foods. (2024) 13:3378. doi: 10.3390/foods13213378. PMID: 39517162 PMC11545549

[29] Abhishek Chakkaravarthi S Agarwal T . Fish consumption patterns and health risk assessment of polycyclic aromatic hydrocarbons and polychlorinated biphenyls in fried and grilled fish products and mitigation strategies. Toxicol Rep. (2025) 14:101953. doi: 10.1016/j.toxrep.2025.101953. PMID: 40612662 PMC12223421

[30] Tongo I Ogbeide O Ezemonye L . Human health risk assessment of polycyclic aromatic hydrocarbons (PAHs) in smoked fish species from markets in Southern Nigeria. Toxicol Rep. (2017) 4:55–61. doi: 10.1016/j.toxrep.2016.12.006. PMID: 28959625 PMC5615098

[31] Rigi P Kamani H Ansari H Mohammadi L Dargahi A . Health risk assessment of polycyclic aromatic hydrocarbon compounds (PAHs) in grilled meats in Zahedan city of Iran. Sci Rep. (2025) 15:24267. doi: 10.1038/s41598-025-03807-w. PMID: 40624067 PMC12234855

[32] Codex Alimentarius . Code of practice for the reduction of contamination of food with polycyclic aromatic hydrocarbons (PAH) from smoking and direct drying processes. Rome: Food and Agriculture Organization of the United Nations (2009). CAC/RCP 68-2009.

[33] Nnaji J Ekwe N . Effect of smoking on polycyclic aromatic hydrocarbons (PAHS) concentrations in catfish and tilapia muscles. J Appl Sci Environ Manage. (2018) 22:293–7. doi: 10.4314/jasem.v22i2.23

[34] Schrenk D Bignami M Bodin L Chipman JK del Mazo J Grasl-Kraupp B . Assessment of the health risks related to the presence of polycyclic aromatic hydrocarbons in some foods. EFSA J. (2022) 20:e07591. doi: 10.2903/j.efsa.2022.7591. PMID: 36381127 PMC9644229

[35] Sampaio GR Guizellini GM da Silva SA de Almeida AP Pinaffi-Langley ACC Rogero MM . Polycyclic aromatic hydrocarbons in foods: Biological effects, legislation, occurrence, analytical methods, and strategies to reduce their formation. Int J Mol Sci. (2021) 22:6010. doi: 10.3390/ijms22116010. PMID: 34199457 PMC8199595

[36] European Commission . Commission Regulation (EU) 2023/915 of 25 April 2023 on maximum levels for certain contaminants in food and repealing Regulation (EC) No 1881/2006. L 119/103: Official Journal of the European Union (2023). Available online at: https://eur-lex.europa.eu/eli/reg/2023/915 (Accessed October 15, 2025).

[37] Adeyeye SAO Ashaolu TJ . A study on polycyclic aromatic hydrocarbon and heavy metal concentrations of commercial grilled meat (Suya) and smoked catfish (Clarias gariepinus Burchell 1822) fish from South-West, Nigeria. Polycyclic Aromat Compd. (2022) 42(6):3281–3290. doi: 10.1080/10406638.2020.1858883. PMID: 41909888

[38] Asamoah EK Nunoo FKE Addo S Nyarko JO Hyldig G . Polycyclic aromatic hydrocarbons (PAHs) in fish smoked using traditional and improved kilns: Levels and human health risk implications through dietary exposure in Ghana. Food Control. (2021) 121:1–9. doi: 10.1016/j.foodcont.2020.107576. PMID: 41909469

[39] Muhammad Abdulla S Ebadi Fathabad A Sadighara P Jamalvandi N Reshadat Z Aslani R . Levels and health risk assessment of polycyclic aromatic hydrocarbons in grilled meat in the Kurdistan region of Iraq. Sci Rep. (2025) 15:31691. doi: 10.1038/s41598-025-16484-6. PMID: 40877437 PMC12394643

[40] Darwish WS Chiba H El-Ghareeb WR Elhelaly AE Hui SP . Determination of polycyclic aromatic hydrocarbon content in heat-treated meat retailed in Egypt: Health risk assessment, benzo[a]pyrene induced mutagenicity and oxidative stress in human colon (CaCo-2) cells and protection using rosmarinic and ascorbic acids. Food Chem. (2019) 290:114–24. doi: 10.1016/j.foodchem.2019.03.127. PMID: 31000027

[41] Nabizadeh S Aeini K Barzegar F Arabameri M Hosseini H Kamankesh M . Volatile N-nitrosamines in processed meat products: An approach for monitoring dietary exposure, assessing human risk, and evaluating variable correlations by principal component analysis and heat map. Food Chem Toxicol. (2024) 188:114649. doi: 10.1016/j.fct.2024.114649. PMID: 38599275

[42] Assogba MF Anihouvi DGH Iko Afé OH Kpoclou YE Mahillon J Scippo M-L . Processing methods, preservation practices and quality attributes of smoked and smoked-dried fishes consumed in Benin. Cogent Food Agric. (2019) 5:1–13. doi: 10.1080/23311932.2019.1641255. PMID: 41909888

[43] Yusuf KA Ezechukwu LN Faykoya KA Akintola SL Agboola JI Omoleye TO . Influence of fish smoking methods on polycyclic aromatic hydrocarbons content and possible risks to human health. Afr J Food Sci. (2015) 9:126–35. doi: 10.5897/AJFS2014.1227. PMID: 38147025

[44] Ekanem AM Ijezie AE Udo IA Ekrikpo UE Idung AU . Meat singeing practices and knowledge of its effects on health and environment among butchers in Uyo, Nigeria. J Adv Med Pharm Sci. (2020) 22:23–33. doi: 10.9734/jamps/2020/v22i730182, PMID: 42045986

[45] World Health Organization . Evaluation of certain food additives and contaminants: seventy-third report of the Joint FAO/WHO Expert Committee on Food Additives. Geneva: World Health Organization (2011).

[46] Dessalegn B Enqueselassie F Kaba M Assefa M Addissie A . Risk factors of oesophageal cancer at health facilities in Addis Ababa, Ethiopia: Unmatched case control study. Front Oncol. (2022) 12:997158. doi: 10.3389/fonc.2022.997158. PMID: 36203447 PMC9530820

[47] United States Environmental Protection Agency . Guidelines for Carcinogen Risk Assessment. EPA/630/P-03/001F. Washington, DC: US EPA, Risk Assessment Forum (2005).

[48] Nsonwu-Anyanwu AC Helal M Khaled A Umoh SI Usoro CAO Eltigani TF . Urinary biomonitoring and cancer risk assessment of polycyclic aromatic hydrocarbon exposure in relation to water intake in Calabar, Nigeria. Exposure Health. (2025) 17:875–86. doi: 10.1007/s12403-025-00704-5. PMID: 41913934

[49] Bwala MN Imam TS Zungum IU . Determination of heavy metals contamination on smoked fish sold at some fish markets in Borno State, Nigeria. J Chem Health Risks. (2023) 13(1):135–43. doi: 10.22034/jchr.2022.1945462.1458

[50] Dada EO Osilagun HO Njoku KL . Physicochemical and genotoxic evaluations of singed cowhide meat (Ponmo) wastewater. J Health pollut. (2018) 8:181207. doi: 10.5696/2156-9614-8.20.181207. PMID: 30560006 PMC6285674

[51] Nebert DW Dalton TP Okey AB Gonzalez FJ . Role of aryl hydrocarbon receptor-mediated induction of the CYP1 enzymes in environmental toxicity and cancer. J Biol Chem. (2004) 279:23847–50. doi: 10.1074/jbc.r400004200. PMID: 15028720

[52] Bray F Ferlay J Soerjomataram I Siegel RL Torre LA Jemal A . Global cancer statistics 2018: GLOBOCAN estimates of incidence and mortality worldwide for 36 cancers in 185 countries. CA: A Cancer J For Clin. (2018) 68:394–424. doi: 10.3322/caac.21492. PMID: 30207593

[53] Uwagba G Joseph A Habeebu M Aje E Oladipo A Fagbemide O . Advanced-technique radiation therapy for nasopharyngeal carcinoma in a low resource setting: a review of treatment-related quality of life. Ecancermedicalscience. (2024) 18:1770. doi: 10.3332/ecancer.2024.1770. PMID: 39430069 PMC11489088

[54] Marcus PM Vineis P Rothman N . NAT2 slow acetylation and bladder cancer risk: a meta-analysis of 22 case-control studies conducted in the general population. Pharmacogenetics. (2000) 10(2):115–22. doi: 10.1097/00008571-200003000-00003, PMID: 10761999

[55] Ndebia EJ Kamsu GT . A comprehensive meta-analysis of dietary and culinary practices on esophageal cancer incidence in the East African Corridor. SVU-International J Med Sci. (2024) 7:207–22. doi: 10.21608/SVUIJM.2024.263853.1785

[56] Iko Afé OH Kpoclou YE Douny C Anihouvi VB Igout A Mahillon J . Chemical hazards in smoked meat and fish. Food Sci Nutr. (2021) 9:6903–22. doi: 10.1002/fsn3.2633. PMID: 34925818 PMC8645718

[57] Lu F Kuhnle GK Cheng Q . The effect of common spices and meat type on the formation of heterocyclic amines and polycyclic aromatic hydrocarbons in deep-fried meatballs. Food Control. (2018) 92:399–411. doi: 10.1016/j.foodcont.2018.05.018. PMID: 41909469

[58] Tian H Yu J Li M Li J Lu Y Yu X . Effect of curcumin on the formation of polycyclic aromatic hydrocarbons in grilled chicken wings. Food Chem. (2023) 414:135561. doi: 10.1016/j.foodchem.2023.135561. PMID: 36827781

[59] Nowar D Edris S Sabeq I . Polycyclic aromatic hydrocarbon mitigation in beef and camel steaks using plant juice and waste marinades. NPJ Sci Food. (2025) 9:141. doi: 10.1038/s41538-025-00501-z. PMID: 40681544 PMC12274582

[60] European Food Safety Authority . Scientific opinion on the safety of pulegone and menthofuran as food flavouring substances. EFSA J. (2016) 14:e04562. doi: 10.2903/j.efsa.2016.4562. PMID: 41290924

[61] Council of Europe . Technical guide on metals and alloys used in food contact materials and articles. Strasbourg: Council of Europe Publishing (2013).

[62] Mali A Laskar SK Das A Upadhyay S Choudhury S Pame K . Effect of different smoking methods and levels of fat on the polycyclic aromatic hydrocarbon (PAHs) in buffalo meat sausages. Indian J Anim Res. (2025) 59:1586–92. doi: 10.18805/IJAR.B-5602

[63] Lu J Hu M Chen X Li Z Nie W Cai K . Visible light-mediated degradation of benzo(a)pyrene in smoked sausages by liposome-encapsulated hydrogenated TiO_2_: Insights into mechanisms and pathways. Food Chem. (2025) 493:145888. doi: 10.1016/j.foodchem.2025.145888. PMID: 40816080

[64] World Health Organization . One Health. Geneva: World Health Organization (2017). Available online at: https://www.who.int/news-room/q-a-detail/one-health (Accessed October 18, 2025).

[65] Kim HJ Cho J Kim D Park TS Jin SK Hur SJ . Effects of Gochujang (Korean Red Pepper Paste) Marinade on Polycyclic Aromatic Hydrocarbon Formation in Charcoal-Grilled Pork Belly. Food Sci Anim Resour. (2021) 41(3):481–496. doi: 10.5851/kosfa.2021.e12, PMID: 34017956 PMC8112319

